# Identifying Metal Binding Sites in Proteins Using Homologous Structures, the MADE Approach

**DOI:** 10.1021/acs.jcim.3c00558

**Published:** 2023-08-09

**Authors:** Vid Ravnik, Marko Jukič, Urban Bren

**Affiliations:** † Faculty of Chemistry and Chemical Engineering, University of Maribor, Smetanova ulica 17, Maribor SI-2000, Slovenia; ‡ The Faculty of Mathematics, Natural Sciences and Information Technologies, University of Primorska, Glagoljaška 8, Koper SI-6000, Slovenia; § Institute for Environmental Protection and Sensors, Beloruska ulica 7, Maribor SI-2000, Slovenia

## Abstract

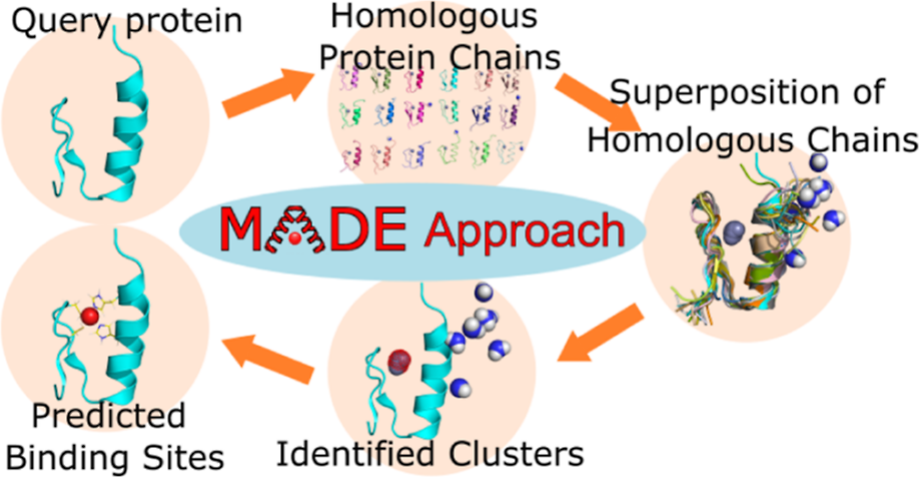

In order to identify the locations of metal ions in the binding sites of proteins, we have developed a method named the MADE (MAcromolecular DEnsity and Structure Analysis) approach. The MADE approach represents an evolution of our previous toolset, the ProBiS H_2_O (MD) methodology, for the identification of conserved water molecules. Our method uses experimental structures of proteins homologous to a query, which are subsequently superimposed upon it. Areas with a particular species present in a similar location among many homologous protein structures are identified using a clustering algorithm. Dense clusters likely represent positions containing species important to the query protein structure or function. We analyze well-characterized *apo* protein structures and show that the MADE approach can identify clusters corresponding to the expected positions of metal ions in their binding sites. The greatest advantage of our method lies in its generality. It can in principle be applied to any species found in protein records; it is not only limited to metal ions. We additionally demonstrate that the MADE approach can be successfully applied to predict the location of cofactors in computer-modeled structures, e.g., via AlphaFold. We also conduct a careful protein superposition method comparison and find our methodology robust and the results largely independent of the selected protein superposition algorithm. We postulate that with increasing structural data availability, additional applications of the MADE approach will be possible such as non-protein systems, water network identification, protein binding site elaboration, and analysis of binding events, all in a dynamic manner. We have implemented the MADE approach as a plugin for the PyMOL molecular visualization tool. The MADE plugin is available free of charge at https://gitlab.com/Jukic/made_software.

## Introduction

Metals play an important role in biological systems; more than a third of all known protein structures contain metal ions.^1^ About 12 different metals are commonly associated with proteins.^2^ The role of metal ions in proteins can be crucial and falls broadly into two categories: structural and functional.^3^ For example, they can stabilize protein structures or even serve as cross-linking agents.^4^ They can also play a functional role by being directly involved in the chemical reactions catalyzed by metalloenzymes,^5^ e.g., serving as redox centers, or as electrophilic reactants that bring reactive groups into the correct orientation.^3^ Additionally, metals can play a regulatory role, for example, in signal transduction.^4^

Due to the abundance and importance of metal ions in proteins, knowledge about their binding is crucial for our understanding of biological systems. Experimental techniques, like X-ray crystallography, NMR spectroscopy, or cryogenic electron microscopy, provide the most reliable information on this topic, but we can quickly run into problems with their cost, time consumption, or automation of the processes.^6,7^ When experimental data are difficult to obtain in a timely manner, we can turn to computational methods which allow for relatively quick and easy identification of metal binding sites. Most of them are based either upon analyzing the amino acid sequence of a given protein or upon finding structural motifs that are analogous with the ones in known, well-characterized metal binding proteins.^7,8^

Examples of algorithms for the prediction of metal binding sites based on sequence homology include SeqCHED Server,^9^ MetalDetector,^10^ IonSeq,^11^ and ZincFinder.^12^ Other tools such as CHED,^13^ FINDSITE-metal,^14^ mFASD,^15^ MIB,^16^ TEMSP,^17^ and FEATURE metal scanning^18,19^ apply structural information for the prediction of metal binding sites.^8^ Some methods combine sequence and structural information, for example, MetSite,^20^ IonCom,^11^ or 3DLigandSite.^21^ Many methods also employ machine learning approaches to predict metal binding,^6^ among these are the already listed IonSeq and IonCom^11^ tools; a recent example on predicting the Ca^2+^ and Mg^2+^ ligand binding with a deep neural network can be found in ref (22).

Many of the above listed software tools limit the range of metal ions they detect by focusing only on specific amino acid residues.^7^ As an example, a well-known algorithm CHED is based on the fact that four amino acid types, Cys (C), His (H), Glu (E), and Asp (D) (referred to as “CHED”), are most commonly involved in metal binding. CHED can predict the binding sites of certain transition metals (Zn, Co, Ni, Fe, Cu, and Mn)^13^ and operates by identifying triads of C,H,E,D residues satisfying geometric criteria from a statistical search of *holo* and *apo* protein pairs.^4,5,13^

We have here devised a novel, simple, and fast approach capable of identifying metal binding sites, called the MADE (MAcromolecular DEnsity and Structure Analysis) approach, which uses structural data and utilizes a superposition of homologous protein structures. Recent advances in experimental techniques such as cryo-EM^23,24^ promise an ever-growing body of experimental protein structures.^25^ This means that we can consider the structural data reliance of the MADE approach beneficial since the predictive power of the methodology will increase with additional structural data. Moreover, we would also like to mention recent advances in protein structure prediction such as DeepMind’s AlphaFold2^26−28^ which offer enormous potential for generating structural data. Currently, AlphaFold and most other computational methods for protein structure prediction do not include the cofactors,^29,30^ so, unfortunately, they cannot help our methodology. However, this in turn means that the MADE approach can also be applied to locate the ionic cofactors in predicted protein structures.

The idea behind the MADE approach comes from the ProBiS H_2_O^31^ (MD^32^) methodology for identifying conserved water molecules in protein structures. ProBiS H_2_O predicts conserved water sites on a query protein by first superimposing homologous structures from the Protein Data Bank (RCSB PDB^33^) onto the query. It applies the ProBiS algorithm for superposition. The next step consists of transposing water molecules from the superposed structures to the common coordinate system of the query. A clustering algorithm (3D-DBSCAN^34,35^) is used to locate dense areas of water molecules, which are identified as conserved water sites and ranked according to the number of occupants in a particular cluster.^31,32^

The MADE method represents a generalization of the ProBiS H_2_O approach: instead of focusing solely on conserved water molecules, we apply a similar workflow to all species present in the query protein and in the homologous structures. This allows us to locate positions where certain species are present among many homologous structures which can be interpreted as indicators of species important for the query protein structure or function.

In the current study, our main focus represents the application of the MADE approach to studying metal ions in protein structures. The approach can locate the binding sites of metal ions in *apo* structures, while in *holo* structures, it can distinguish metals important for the structure from metal ions only present as impurities. We have successfully used the approach to predict the locations of metal ion binding sites in *apo* protein structures, and we present the prediction capabilities of the approach as the main message of this article.

The method is implemented as the MADE plugin for the PyMOL Molecular Graphics System^36^ (v2.6), which facilitates a user-friendly interface as well as a quick visualization of the results. The MADE plugin is freely available at https://gitlab.com/Jukic/made_software, and we are fully committed to supporting this toolset in the future.

In the Methods section, we describe the MADE approach and its implementation as the MADE plugin in more detail. In the Results and Discussion section, we demonstrate its application to some well-characterized metalloprotein examples. We also compare the results of the approach when using different superposition methods.

## Methods

The developed MADE approach applies homologous protein superposition, followed by a clustering algorithm to predict the locations of important species in a given protein structure. It can be utilized to predict the locations of metal binding sites.

**Figure 1:**
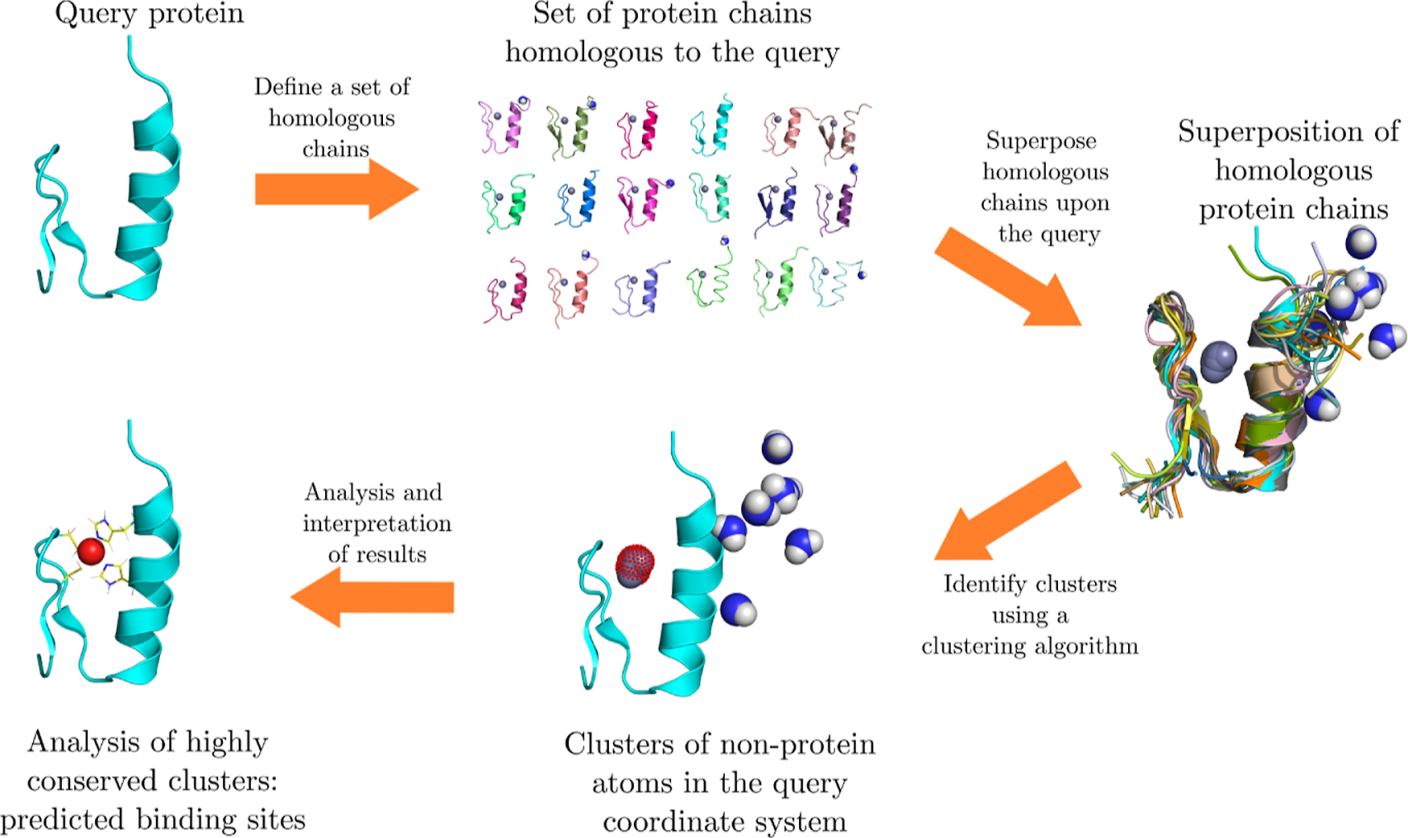
MADE approach: first, we identify a set of protein chains homologous to our query, these are then superimposed onto the query protein, and next dense areas of a particular species are located with a clustering algorithm. Clusters with many members likely represent species important to the query protein structure.

Figure 1 depicts the steps of the MADE approach. We start with the structure of a query protein we want to analyze. The first step of our methodology represents identifying a set of protein chains similar to the query. We are interested in locations where a particular species is located in a similar position among many homologous protein structures. In order to locate such areas, the next step applies a superposition algorithm to superimpose the homologous protein structures upon the query. We keep track of the locations of all the atoms in the superposed homologous protein structures using the common coordinate system of the query. The subsequent step applies a clustering algorithm to identify dense areas of a particular species. The average locations of the members of the identified dense clusters reveal the desired positions, which can be interpreted as likely containing a species of importance to the original query protein structure.

We present metal ions as an example: if a metal ion is important to the functioning of a given protein structure, many of the homologous protein structures will likely also include this ion in their binding site. Therefore, our approach will locate a dense cluster of metal ions in the binding site of the query protein. On the other hand, if metal ions are not usually present at a particular location in the protein structure, e.g., if they are present as impurities only, then they will not have corresponding ions in a similar position in many homologous protein structures; therefore, no dense cluster will be found. In this way, our approach can distinguish metal ions crucial to the protein structure from the less important ones.

In this paper, we focus on the MADE approach as a tool to detect metal binding sites, but in principle, the approach can be applied to any species in protein structural records and is by no means limited to metals. For example, the MADE approach retains the conserved water molecule prediction capabilities of ProBiS H_2_O^31^ upon which it is based. Our implementation of the MADE approach as the MADE plugin also expands on the ProBiS H_2_O tool by offering alternative methods for protein superposition before the data analysis step. In the following sections, we will describe the MADE approach and its implementation as the MADE plugin in more detail.

### Identification of Homologous Protein Structures

The first step in the MADE approach represents defining a set of protein structures homologous to the query protein. Our implementation in the MADE plugin uses experimental protein structures from the Protein Data Bank RCSB PDB.^33^ In the MADE plugin, the user can select the query protein structure by inputting its PDB accession code or by providing a custom .pdb file. Moreover, the plugin also supports structures predicted by AlphaFold2, accessible to the user by inputting the CMS ID of the structure on the RCSB PDB. After defining the query, the subsequent step of the MADE approach represents identifying a set of homologous protein structures.

We utilize the pre-calculated sequence identity clusters provided by the RCSB PDB.^37^ These represent groups of protein structures sharing at least a preset amount of sequence identity. They are given as plain text files for different sequence identity cutoffs with a cluster of polymer entities per line.^38^ As of April 2022, these files contain polymer entity identifiers instead of chain identifiers to avoid redundancy.^37,39^ Currently, the clustering algorithm MMseqs2^40^ is applied to obtain the sequence similarity clusters on the PDB website.

In the MADE plugin, the user can select the sequence identity cutoff (30, 40, 50, 70, 90, 95, or 100%). The plugin finds the line containing the query protein PDB ID in the sequence identity cluster file for the appropriate cutoff and subsequently queries the PDB for the protein chains belonging to each of the polymer entities from the sequence identity cluster. Alternatively, the user can provide a custom set of homologous protein structures using a file formatted in an analogous manner to the sequence identity cluster files from the PDB. In this study and in the MADE plugin, we selected homologs to the query protein based on sequence identity, but this is not the only option. We believe that (local) structure similarity could be a sufficient criterion to find homologous structures for the MADE approach. Tools exist which can search for structures sharing structural similarity with the query protein; the PDB can search for globally similar structures,^41^ while other tools can find structures sharing only a similar local structure, for example, the ProBiS web server.^42,43^

### Superposition of Protein Chains

After defining the set of homologous protein chains, their superposition upon the query protein structure follows. The MADE plugin offers a choice of multiple algorithms for protein superposition, namely, the *align*(44,45) and *super*(46,47) commands in PyMOL, TM-align,^48^ DeepAlign,^49^ GANGSTA+,^50^ and ProBiS,^51^ which are described in more detail below.

The MADE plugin can superimpose the homologous chains on the whole protein chain of the query or on a local binding site around a group present in the query protein structure. Local superposition often yields better results for species in the vicinity of the binding site of the query. For example, local superposition can be used when we are interested in species around a particular binding site, e.g., conserved water molecules around an ion present in the query protein structure. Local superposition requires additional input from the user about the binding site they wish to examine. On the other hand, whole chain superposition provides data about species in the entire protein chain, although the results tend to be harder to interpret due to the abundance of information. For all the superposition methods except ProBiS, the MADE plugin achieves local superposition by creating a new structure composed of only residues from the query protein within a certain radius of the selected binding site (the radius is configurable in the MADE plugin, 12 Å was applied in this study). The plugin then superposes the homologous structures with the residues near the binding site. ProBiS represents the exception, local superposition is its specialty, and it has a built-in local superposition mode that the MADE plugin applies.

#### PyMOL *Align*

The *align* command of PyMOL represents a superposition algorithm based on a dynamic programming sequence and its subsequent iterative refinement. The *align* command initially performs a global BLAST-like BLOSUM62-weighted dynamic programming sequence and then establishes a per-atom correspondence between selections. Matching side chains are included. An initial superposition is then performed, followed by an iterative refinement, where atoms with high per-atom deviations are thrown out and the fit is repeated.^44,45^

#### PyMOL *Super*

Unlike the *align* command, the *super* command in PyMOL performs a sequence-independent dynamic programming alignment. This is followed by a series of iterative refinement cycles, similar to the *align* command. It is reportedly more robust than the *align* algorithm for proteins sharing a low sequence similarity.^46,47^

#### TM-Align

TM-align represents a sequence-independent algorithm for protein superposition. TM-align only employs the coordinates of the *C*_α_ atoms of the given protein structures, it does not concern itself with the protein side chains when calculating the superposition. TM-align first obtains an initial alignment, which is then subjected to a heuristic iterative algorithm. The optimal alignment is based on the TM-score, which assigns a bigger weight to residue pairs at small distances than to residue pairs far apart. This means that the TM-score would not unduly penalize structures with a very similar global topology but with a few larger local deviations.^48^

#### DeepAlign

DeepAlign represents a protein alignment tool using both sequence and evolutionary information. The DeepAlign algorithm identifies similar fragment pairs that are evolutionary-related using local structure substitution and amino acid matrices. The initial alignment is generated from one of these fragment pairs and is further refined through dynamic programming and gap elimination. The scoring function which determines how likely two residues are to be aligned, is composed of geometric similarity (TM-Score), hydrogen bonding similarity, amino acid mutation score, and local substructure substitution similarity. To calculate the TM-score, the coordinates of C_α_ atoms are again used.^49^

#### GANGSTA+

GANGSTA+ represents a protein structure alignment tool that initiates the protein superposition by aligning the secondary structure elements (SSEs, α helices, and β sheets) of the proteins while ignoring the loops connecting them. In the subsequent stage, the residue pair assignment is performed on the basis of the results of the SSE alignment. Finally, a refinement of the residue pair assignment is applied to complete the SSE assignment from the initial stage as well as to perform reassignments of SSEs and to extend the residue pair assignment beyond the SSE boundaries. Due to initially ignoring the loops connecting SSEs, GANGSTA+ can align proteins non-sequentially. Protein alignments in GANGSTA+ are evaluated using the structure alignment score, which weighs the RSMD of C_α_ atoms relative to the number of aligned residues.^50^

#### ProBiS

The ProBiS algorithm facilitates local superposition of the surfaces of different proteins. Protein structure comparison includes geometrical and physicochemical properties of amino acid residues of the protein structure. The ProBiS algorithm detects structural patches that are common to both protein surfaces by interpreting surface amino acid residues as vertices in the 3D space, where a structurally similar site is the one with a similar arrangement of vertices. ProBiS applies a fast maximum clique algorithm^52^ to detect similar protein surface patches. ProBiS does not attempt to align proteins globally but exclusively around a similar patch. Therefore, a similar folding is not a prerequisite for a similarity between two protein structures.^51,53^ ProBiS also offers a web server capable of comparing the surface of a query protein with a database of nonredundant protein structures based on the PDB and detects similar sites.^42^ Both the web server and the ProBiS algorithm have received numerous updates through the years,^43,54−57^ including a database of precalculated binding site similarities and local alignments of PDB structures.^58^ Recently, the ProBiS-Fold approach has been developed, it can annotate structures from the AlphaFold database lacking corresponding structures in the PDB, with the goal of discovering new druggable sites.^93^

### Clustering

After protein superposition, the MADE approach applies a clustering algorithm to detect dense areas where a particular species is present in many of the homologous protein chains. Our implementation of the MADE plugin is designed to work with .pdb files and focuses on entries marked as HETATM. PDB files are slowly being replaced by PDB Exchange/Macromolecular Crystallographic Information (PDBx/mmCIF) Files,^59^ which also features HETATM entries for compatibility with the old .pdb format. Therefore, we intend to extend our support to this file format in the future.

HETATM entries represent non-polymer or other non-standard species in a given protein structure; we will refer to these as “heteroatoms”. The plugin keeps track of the positions of all the heteroatoms from the superimposed chains in the common coordinate system of the query protein. Heteroatoms are assigned a type based on the residue they originate from and the type of the atom. As an example, a water molecule has the residue name HOH in .pdb files, and the oxygen in the water has the atom name O. The MADE plugin would, therefore, denote the type of oxygen in water as HOH–O.

Next, the plugin applies a clustering algorithm on the coordinates of all the heteroatoms of a specific type. The MADE plugin uses the Three Dimensional Density Based Spatial Clustering of Applications with Noise (3D-DBSCAN^34,35^) algorithm implemented in the scikit-learn Python machine learning library.^60^ We have chosen 3D-DBSCAN because it has proven effective for a similar application in ProBiS H_2_O.

DBSCAN represents a clustering algorithm that applies a simple minimum density level estimation based on two parameters, *n* and ϵ. Points with more than *n* neighbors (including the initial point) within a radius ϵ are considered *core points* of a cluster. All points within a radius ϵ are considered a part of the same cluster as the core point (*direct density reachable*). If any of the neighbors are themselves core points, their neighbors are also transversely included (*density reachable*). Points in a cluster that are not core points are called *border points*.^34,35^

The radius ϵ can be defined in the MADE plugin; throughout this study, a value of ϵ = 0.9 Å was used. In the MADE plugin, the minimum number of neighbors parameter *n* is iteratively increased from 1 to *N*_sp_, where *N*_sp_ is the number of superimposed protein structures.

The result of the MADE plugin represents a list containing detected clusters of different types of heteroatoms, with different minimum numbers of members calculated by an iterative increase in *n*. The plugin lists the lowest conservation in a group of clusters; as an example, the plugin may list 1 cluster of ZN–ZN (zinc ion, residue name ZN, atom name ZN in the PDB files) ions with 95% conservation and 2 clusters of ZN–ZN with at least 85% conservation. This means that the plugin located 1 cluster in which 95% of the superimposed protein structures exhibit a Zn ion in a similar position and 2 clusters where at least 85% of the superimposed structures have a Zn ion in a similar position, including the previous 95% preserved cluster.

Cluster conservation is defined as the number of heteroatoms of a specific type in a given cluster divided by the total number of superimposed protein structures (Equation 1).
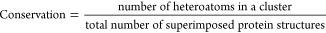
1

A high conservation value tells us that the specific heteroatom is present in a similar location in many of the superimposed structures; therefore, it is likely to be relevant to the query protein structure.

### Analysis of the Results from the MADE Approach

The main result of the MADE approach represents a list of clusters of various species with different degrees of conservation. We calculate the average position of members of a particular cluster, which represents the position of the cluster. The analysis and interpretation of the clusters is left to the user, where highly conserved clusters are treated as indicators of important species which merit further mechanistic or structural investigation.

In this work, we focused on detecting metal binding sites; therefore, we analyzed our results by searching for highly conserved clusters of metal ions. These likely represent the positions of the metal ions in their binding sites. Finding other species, e.g., conserved water molecules, which are also important for describing the metal binding site can be more difficult. We recommend making use of local superposition since it filters out most of the information about species not present in the binding site. Further analysis can be conducted by studying the nearby amino acid residues.

The MADE plugin offers tools to make the analysis of results easier; the main one represents the ability to display the location of a given cluster in the PyMOL viewer. The MADE plugin can also easily display the amino acid residues surrounding the displayed cluster, as well as calculate and visualize the distances to the closest amino acid residues. When performing whole chain superposition, the amount of information can be overwhelming. The MADE plugin, therefore, offers certain options to filter the list of the results for specific species only.

It is difficult to determine a singular cutoff for the cluster conservation after which we treat clusters as potentially relevant to the query protein structure. Even clusters with very low conservation can turn out to be structurally important to the query. As an example, we find a 15% conserved cluster of OH^–^ ions when analyzing the *apo* manganese superoxide dismutase structure in the subsequent chapter. We demonstrate that this OH^–^ shares a position coordinating the Mn ion with better conserved cluster of water molecules, showing us that Mn is sometimes coordinated by OH^–^, which is also confirmed by the scientific literature.^61,62^

### Implementation and Visualization

**Figure 2:**
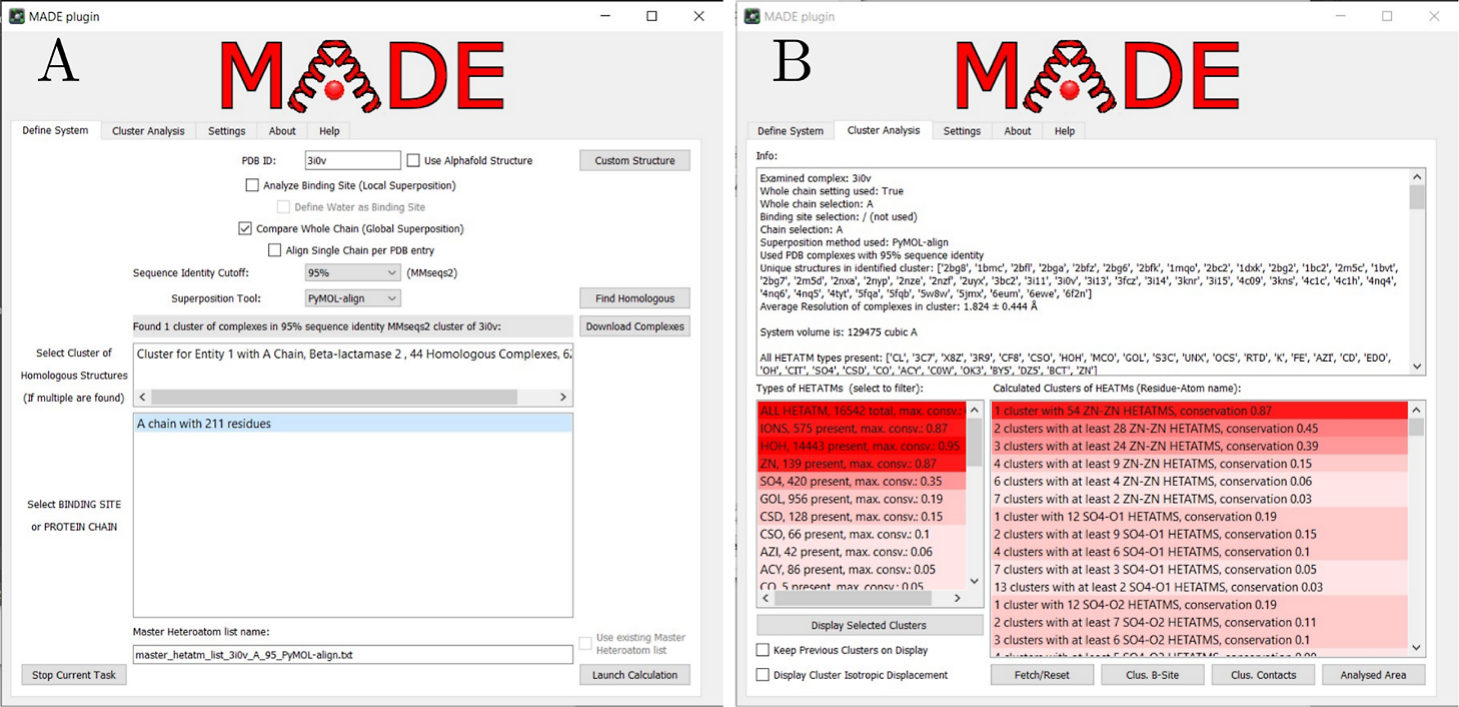
MADE plugin. (A) Define System tab with the PDB ID input field (or Custom Structure for inputting a custom .pdb file). The Use Alphafold structure check allows the use of a protein structure modeled by AlphaFold2. Underneath we specify, if we want to analyze a whole protein chain or a specific binding site, and if we want at most one chain per PDB entry. Below one finds a dropdown list that allows them to choose a sequence identity cutoff for the MMseqs2 clustering data (Or a custom cluster of complexes). There is also a dropdown list to select between different superposition methods. The Find Homologous button finds the set of homologous structures based on the query protein PDB ID and on the chosen sequence identity cutoff. If the query consists of multiple chains with different clusters of homologous structures, select the cluster to use in the Select Cluster field. The Download Complexes button downloads the .pdb files from the PDB database if they are not already present. When the query protein structure is downloaded, the user selects a protein chain or a binding site from the Select BINDING SITE or PROTEIN CHAIN list and runs the calculation with the Launch Calculation button. (B) After the calculation finishes, the user is redirected to the Cluster Analysis tab. Here, one finds the Info panel with the information about the parameters of the calculation, as well as about the identities and chemical formulae of heteroatoms located in the analyzed system (taken from the HETNAM and FORMUL entries of the .pdf files). Below on the left is the Types of HETATMs list, which displays the total number and maximum conservation of clusters of all types of heteroatoms present. To the right lies the Calculated Clusters field, which lists all the different clusters of heteroatoms identified. Selecting an entry from the Types of HETATMs list will filter the Calculated Clusters field to display only clusters matching the selected species. Below the Calculated clusters field are buttons used for displaying the results. The Fetch/Reset button will clear the PyMOL display and fetch the query protein structure. To the left, the Display Selected Clusters button will draw the selected clusters as spheres in the PyMOL viewer. The Clus. B-Site button will show the residues around the already displayed clusters, the Clus. Contacts button will show the distances between clusters and the nearby amino acid residues. Last but not least, the Analysed Area button will draw a box around the area in which the plugin searched for clusters.

The MADE approach is implemented as a plugin of the PyMOL Molecular Graphics System^36^ (v2.6), which allows us to easily visualize the obtained results. The plugin is written in python 3; we do not intend to offer official support for python 2. The plugin is supported on the Windows and Linux operating systems (Support might be extended to macOS in the future). A typical workflow with the MADE plugin is as follows: the user must first define the query protein structure (PDB ID or a custom .pdb file), as well as the set of homologous protein structures (sequence identity cutoff or a custom group of structures). The plugin then superimposes the homologous structures upon the query protein and identifies clusters, which are listed and color-coded according to their conservation. The plugin can visualize the average positions of clusters as spheres in the PyMOL viewer. Upon visualizing a given cluster, the plugin also produces a report file with the exact positions and origins of heteroatoms in the cluster. The plugin also provides options to quickly analyze the area surrounding the cluster in the PyMOL viewer by showing the nearby amino acid residues and their distances from the cluster. The MADE plugin GUI is presented in Figure 2, while a more detailed tutorial for the MADE plugin is included in the Supporting Information.

## Results and Discussion

### Identification of Metal Ions in Protein Structures

In this section, we will demonstrate the capabilities of the MADE approach by applying it to several examples. We will focus on well-known metalloproteins, especially on their *apo* forms. We will identify and determine the locations of the metal ions and compare the results of our approach with the scientific literature data on the binding sites of these proteins. Additional information about the examined systems, as well as two additional examples, is available in the Supporting Information. The additional examples consist of a calpain^63^ structure containing calcium ions and a phosphodiesterase^64,65^ containing magnesium and zinc ions.

#### Zinc Finger

Our first example represents a zinc finger superstructure, which forms a common domain found in proteins of eukaryotic organisms.^66^ Zinc fingers are small, functional, and independently folded domains that are coordinated with one or more zinc ions, which stabilize their structure.^67^ In the human genome, around 1000 genes encode proteins containing zinc fingers. Their function ranges from interacting with DNA, RNA, and even with other proteins.^68^

An important family of zinc fingers represents the Cys_2_His_2_ (C_2_H_2_) family, which contains a 28–30 amino acid sequence including two conserved cysteine and two conserved histidine residues.^69^ In the presence of zinc, these sequences form a characteristic compact ββα domain, where the Zn ion is located between the two stranded anti-parallel β-sheet and the α-helix. The zinc is coordinated by the previously mentioned conserved Cys and His residues.^66^ Due to their prevalence, zinc fingers have been an important topic of research.

A study (ref (70)) provides us with the protein structure we analyzed using the MADE approach with the PDB accession code 1RIK.^70^1RIK is of particular interest to our investigation because its PDB record does not contain a zinc ion, which allows us to clearly demonstrate the capabilities of our approach by successfully predicting its location. The study reporting the 1RIK structure focused on designing inhibitors of the E6 protein of papillomavirus. The researchers used the structure of a C_2_H_2_ zinc finger as a scaffold and modified it with a pattern of Leu residues that served as the E6-binding motif. The 1RIK structure consists of a single zinc finger domain 29 residues long,^70^ and further Zn ion location elaboration remains essential.

In order to analyze the 1RIK structure with the MADE approach, we utilized the MADE plugin in a simple and straightforward way. The first step of our approach represents defining a set of protein structures homologous to 1RIK. We applied a 30% sequence identity cutoff, and the sequence clustering data from the RCSB PDB yields a set of 18 complexes homologous to 1RIK. The 1RIK structure has been heavily modified and does not have many homologous structures with a higher sequence identity.

**Figure 3:**
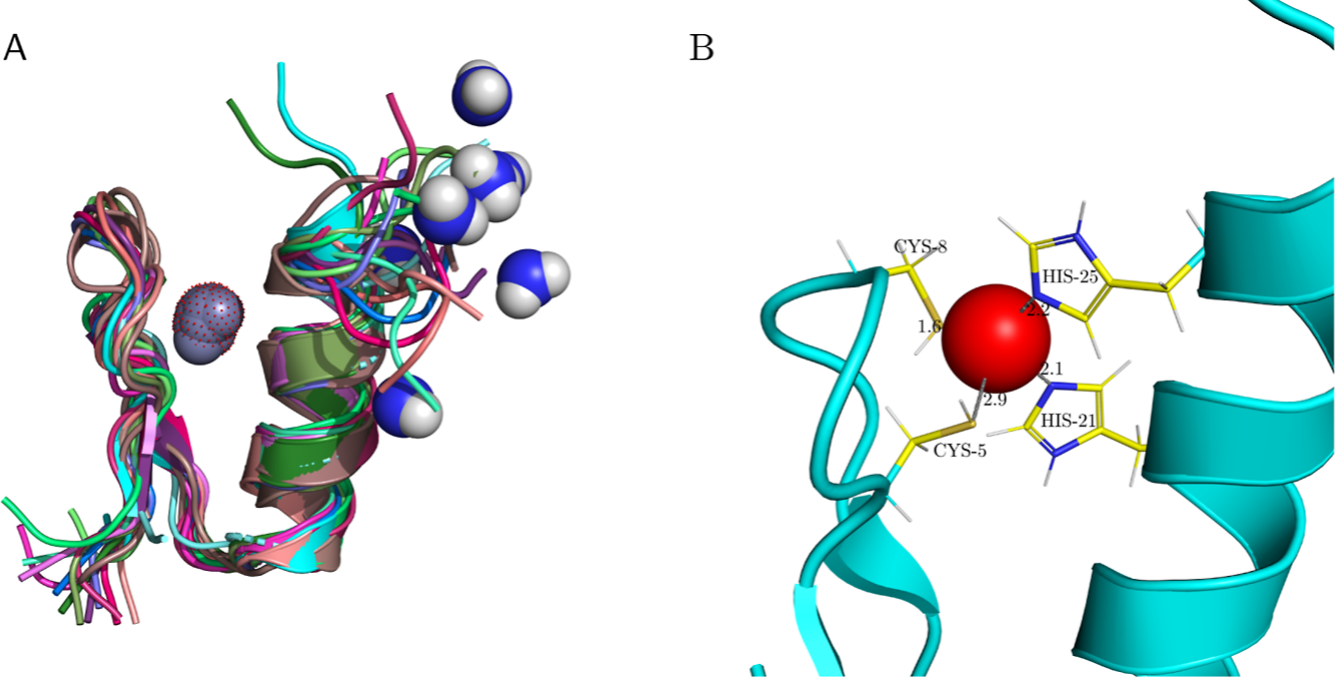
Zinc Finger with PDB ID 1RIK analyzed with the MADE plugin. (A) All the homologous protein structures are superimposed (using DeepAlign^49^) upon the original 1RIK structure (cyan). An isotropic displacement of zinc ions (light purple spheres) in the cluster identified by DBSCAN is displayed with red dots. (B) Cys_2_His_2_ biding site of 1RIK with the calculated average position of the Zn cluster (red sphere). Numbers represent coordinate bond distances in Å.

After defining a set of similar protein chains, the next step represents superimposing them on the original query protein structure. Figure 3A displays the result of this step, all the homologous protein chains superimposed upon 1RIK (superposition with DeepAlign^49^), where the Zn ions are shown as light purple spheres. After superposition, we apply a clustering algorithm to identify dense areas of heteroatoms in the superposed structures. Using 3D-DBSCAN, we locate a cluster of 16 Zn ions (18 total protein chains including 1RIK, conservation of 0.89), where Figure 3A also displays an isotropic displacement of ions in the cluster as red dots. Out of all 18 homologous structures, only the original query protein, 1RIK, does not contain a Zn ion. The Zn ion from one of the superimposed chains is not included in the cluster identified using the selected criteria (ϵ = 0.9 Å) with DBSCAN.

We obtained an approximate location of the Zn ion in the 1RIK structure at position −4.75, 0.62, 1.25 Å. These coordinates were calculated as the average position of the Zn ions in the cluster. The closest protein atoms from the predicted position are the nitrogen atoms of HIS-21 and HIS-25 at a distance of 2.1 and 2.2 Å, respectively, and the sulfur atoms of CYS-5 and CYS-8 at a distance of 2.9 and 1.6 Å, respectively. Figure 3B depicts the predicted Zn ion location with a red sphere while also highlighting the residues forming the Cys_2_His_2_ binding site of 1RIK.

In this simple example, we have shown that the MADE approach can identify a highly conserved cluster of Zn ions in the binding site where one would indeed expect a Zn ion in the protein structure. Further informed structural research should employ additional MD experiments on these starting complexes to obtain optimized systems for further research on the metal coordination geometry. In the rest of this section, we will focus on larger proteins and display that our approach can analogously predict their metal ion binding sites.

#### Manganese Superoxide Dismutase

The next example represents superoxide dismutases (SODs). SODs protect cells from oxidative stress by catalyzing the dissociation of superoxide to hydrogen peroxide and oxygen.^61,71^ SODs are metalloenzymes, often containing Cu, Zn, Fe, or Mn ions.^72^ In this example, we focus on a manganese superoxide dismutase (MnSOD), which is found in most aerobic organisms.^62^ It is usually merged into multimers (dimers or tetramers) of identical subunits. The particular structure we focus on is an *apo*-MnSOD with the PDB accession code 3OT7,^71^ which represents an *Escherichia coli* MnSOD without the manganese cofactors present in its binding site. The crystal structure was obtained in a study (ref (71)) on metal binding into MnSOD using X-ray diffraction at 1.9 Å resolution.

**Figure 4:**
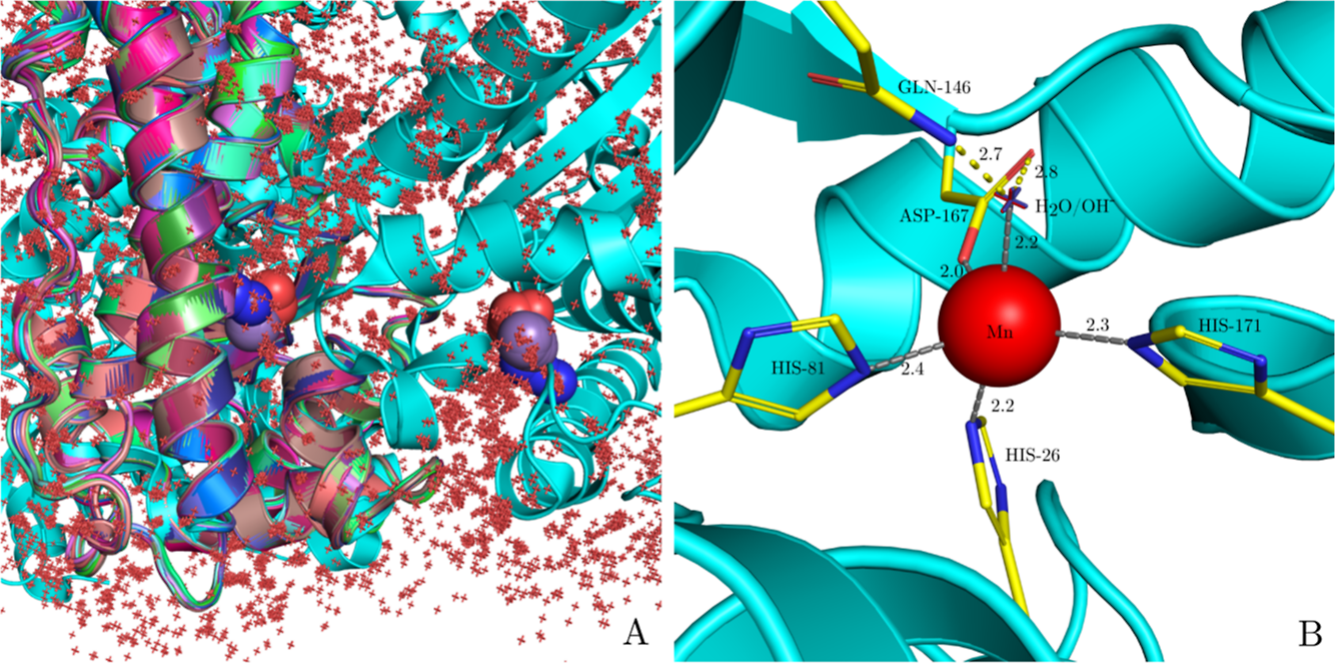
MnSOD structure with PDB ID 3OT7 analyzed with MADE plugin. (A) All homologous structures superimposed (using DeepAlign) upon the query 3OT7 structure (cyan). We see two clusters of Mn ions (light purple spheres) in the binding sites of the A and B chains. (B) Predicted Mn binding site in chain A of 3OT7: position of the cluster of Mn ions predicted by MADE (red sphere) as well as clusters of H_2_O and OH^–^ species (red and blue cross markers, respectively). The coordination of the predicted Mn ion is trigonal bipyramidal (gray lines), and the clusters of H_2_O or OH^–^ can form hydrogen bonds with nearby residues (yellow dotted lines). Numbers display bond distances in Å.

We applied the MADE approach to identify the Mn binding sites in the structure of the *apo*-MnSOD 3OT7. To identify homologous structures, a sequence similarity cutoff of 95% was used. This yielded a set of similar structures containing 13 unique PDB IDs. Many of these encompass multiple homologous chains for a total of 36 superimposed chains upon the query, the chain A of 3OT7. Figure 4A displays the results of the superposition step: all homologous chains superposed upon the query chain A of 3OT7 (cyan) (using DeepAlign) as well as all the heteroatoms in the common coordinate system. 3OT7 forms a much larger system than 1RIK, and there are many more heteroatoms present. The most common ones are water molecules, shown as red cross markers. We are interested in Mn ions, which are depicted as light purple spheres. We can spot dense areas of Mn ions in both chain A as well as in the nearby chain B. Using a clustering algorithm confirms our assertion; DBSCAN reveals two clusters of Mn ions with 87% conservation. The clusters are located in the A and B chains of 3OT7 in the respective binding sites of the subunits.

The binding site of Mn in the 3OT7 chain A is displayed in more detail in Figure 4B. The predicted location of the Mn ion (the average position of Mn ions in the cluster) is visualized as a red sphere at position 33.44, 14.70, 9.44 Å. The closest atoms around the predicted Mn position are the oxygen atom of ASP-167 at a distance of 2.0 Å and the nitrogen atoms of HIS-26, HIS-171, and HIS-81 at a distance of 2.2, 2.3, and 2.4 Å, respectively. Besides the Mn cluster, the MADE plugin allows us to also identify two additional important clusters, a cluster of H_2_O molecules (at position 33.89, 16.69, 10.33 Å) and a cluster of OH^–^ ions (at position 33.87, 16.71, 10.34 Å) with 74 and 15% conservation, respectively. They are located at nearly the same site as can be seen in Figure 4B (red and blue cross markers). The predicted position of the H_2_O molecule and the OH^–^ ion is 2.2 Å away from the predicted position of Mn; close to it, we can also find the nitrogen atom from GLN-146 at a distance of 2.7 Å and the oxygen atom from ASP-167 2.8 Å away. The literature data^61,62^ informs us that the binding of Mn in MnSOD is trigonal bipyramidal, where Mn is coordinated by four residues (three His and one Asp) and one solvent molecule, which is either a water molecule or a hydroxide ion.^61,62^ The clusters found by the MADE approach are indeed in excellent agreement with these requirements, as can be seen in Figure 4B. The Mn cluster is close to His-81, His-26, His-171, and Asp-167. Alongside these residues, the clusters of water molecules and OH^–^ ions identified by the MADE approach form a trigonal bipyramid around the Mn cluster. The distances between the predicted Mn position and the coordinating groups are short, around 2.2 Å. The solvent molecule forms hydrogen bonds with the Gln-146 and Asp-167 residues,^61^ which can also be inferred from our results: the distance between the predicted position of H_2_O/OH^–^ and Gln-146 or Asp-167 is longer (around 2.8 Å), suggesting hydrogen bonding.

The clusters of Mn and solvent molecules identified by the MADE approach describe the binding site of MnSOD well using an *apo* structure. In this example, we can nicely see the advantage of the generality of our approach; beside the location of the Mn ion, a careful analysis of other species has revealed an additional molecule coordinating Mn, a solvent species that is either a conserved water molecule or a hydroxide ion. Conserved water molecules can indeed be crucial for describing the metal binding site of a protein,^31^ as we have demonstrated in this example, and will further show in additional examples.

The article describing the 3OT7*apo* MnSOD structure^71^ notes that the *apo* form of MnSOD is similar to the *holo* form: the binding of Mn does not have a large effect on the conformation of the protein. This similarity helps our approach predict the binding site of Mn so well.

#### Metallo-β-Lactamase

Bacterial resistance to antibiotics represents a major public health threat nowadays.^73−75^ β-Lactam antibiotics are widely prescribed for treating bacterial infections. Bacterial resistance occurs by a number of mechanisms, most commonly by β-lactamases.^74,75^ β-Lactamases represent enzymes that catalyze the hydrolysis of the amide bond in the β-lactame ring and form products with no antibacterial effects.^74^ Class B β-lactamases require Zn ions in their active site to function; they are also known as metallo-β-lactamases (MβLs).

We used the MADE approach on an *apo* metallo-β-lactamase structure, namely, BcII. BcII represents the MβL from *Bacillus cereus*, a typical enzyme of the B1 MβL family. Its *apo* structure with 1.6 Å resolution, PDB accession code 3I0V,^75^ was determined in a study (ref (75)) about metal binding to B1 MβLs.

**Figure 5:**
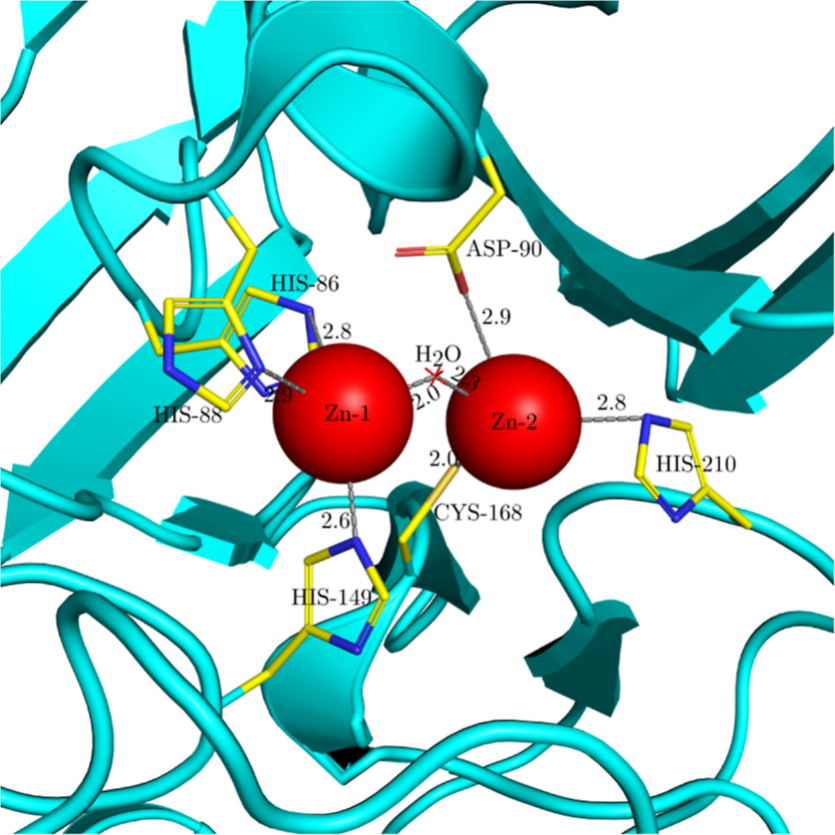
BCII MβL structure with the PDB accession code 3I0V analyzed with the MADE plugin. The positions of the clusters of Zn ions predicted by the MADE approach (red spheres) are shown as well as a cluster of H_2_O molecules (red cross marker). Numbers represent bond distances in Å. The coordination of Zn-1 in the 3H binding site is tetrahedral as expected (three His residues and the conserved bridging water cluster). The expected coordination of Zn-2 is trigonal bipyramidal (DHC, the conserved bridging water molecule and another conserved water molecule), our approach, however, does not find a second water cluster that should coordinate Zn-2.

Using the MADE plugin to predict the Zn binding sites in 3I0V with a 95% sequence identity cutoff, we find a set of 44 homologous complexes totaling 62 protein chains superimposed upon 3I0V. Analyzing the results, we observe three relevant clusters, two clusters of Zn ions (Zn-1 and Zn-2), and one cluster of water molecules. Figure 5 depicts the binding site of 3I0V with the predicted positions of the Zn ions and the water molecule.

Clustering with DBSCAN locates the Zn-1 cluster with 86% conservation at position 6.96, 7.07, 25.99 Å. The second Zn cluster (Zn-2) is much less conserved, with a conservation of 44%, at position 10.14, 7.63, 23.80 Å. This reflects the fact that some reported BcII structures have only a single Zn ion in their active site, specifically in the binding site of Zn-1.^74−78^ This binding site is known as the 3H binding site because Zn-1 is coordinated with three His residues.^74−78^ This also holds for the Zn-1 position predicted by the MADE approach because Zn-1 is close to three His residues: the distance between Zn-1 and His-86, His-88, and His-149 is 2.8, 2.9, and 2.6 Å, respectively.

The second zinc binding site is the DCH binding site because the zinc is coordinated by Asp, His, and Cys residues.^74−78^ The predicted position of Zn-2 is indeed coordinated with Asp-90, His-210, and Cys-168 at a distance of 2.9, 2.8, and 2.0 Å, respectively. The scientific literature tells us that both Zn ions are also coordinated with a water molecule located between them.^74−78^ Clustering with DBSCAN indeed finds a cluster of bridging water molecules in the appropriate location, with 58% conservation (at position 7.99, 8.12, 24.57 Å), also depicted in Figure 5. The predicted conserved bridging water molecule is 2.0 Å away from the predicted Zn-1 and 2.3 Å away from the predicted Zn-2.

Using the identified clusters, we can reproduce the tetrahedral geometry of the 3H (Zn-1) binding site well. On the other hand, studies^74−78^ report that Zn-2 in the DCH site is coordinated in a trigonal bipyramidal configuration with an additional water molecule. However, the results of our conserved water analysis do not corroborate this observation.

The conformations of the amino acid side chains that participate in the metal binding are significantly different in the *apo* and *holo* structures of BcII.^75^ This leads to the distances between the predicted Zn locations and the coordinating residues being larger than expected. González, Buschiazzo, and Vila.^75^ cite metal coordination bond distances for similar BcII systems in the range of 2.2–2.7 Å. Despite the significant differences in the binding site residues of the *holo* and *apo* structures of BcII, the MADE approach is still able to describe metal binding in 3I0V well.

#### Copper Amine Oxidase

Using the MADE approach, we also examined the copper containing amine oxidase from *Arthrobacter globiformis* (AGCO). The selected structure (PDB accession code 3X42) originates from a study (ref (79)) on the effects of halide ions on the catalytic mechanism of the enzyme, which is consequently immersed in a NaBr solution, therefore containing numerous Na^+^ and Br^–^ ions usually not present in the enzyme.

Amine oxidases are responsible for the oxidative deamination of amines to aldehydes. Copper-containing amine oxidases (CAOs) represent a subclass of amine oxidases that are only active against primary amines and contain one of the two quinone-based cofactors, 2,4,5-trihydroxyphenylalanine quinone (topaquinone or TPQ) or lysyl tyrosylquinone. TPQ-containing CAOs produce the cofactor in situ through a post-translational modification of an endogenous amino acid side chain.^80,81^ The studied AGCO contains a TPQ cofactor.

We analyzed the AGCO structure with the PDB accession code 3X42(79) using the MADE approach, and with a 95% sequence similarity cutoff, we find 87 homologous complexes. Most of these structures are homodimers, containing 2 chains, for a total of 153 superimposed protein chains upon the query, chain A of 3X42.

**Figure 6:**
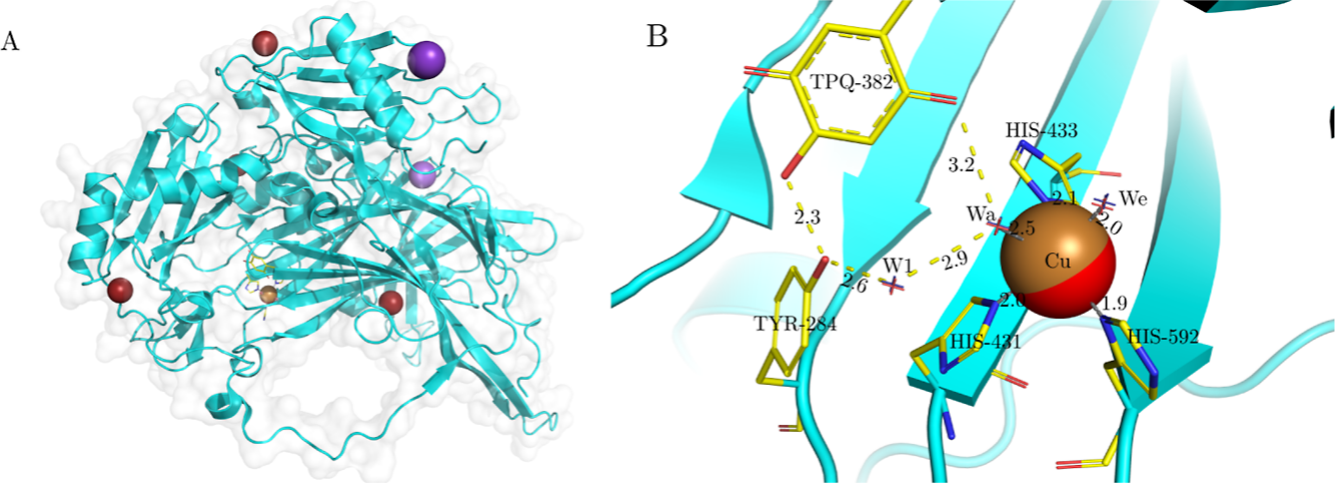
Chain A of AGCO structure with the PDB accession code 3X42 analyzed using the MADE plugin. (A) From the whole protein chain, we can observe many solvent ions. (B) Cu binding site of 3X42, the Cu ion (brown sphere) from 3X42, and the Cu cluster from the MADE approach (red sphere). Three conserved water molecules, Wa, We, and W1 (red cross markers in 3X42, blue cross markers from MADE), are located in the binding site. Hydrogen bonds are indicated with yellow dotted lines and numbers represent bond distances in Å. The Cu ion is bound with a distorted square-planar geometry with three His residues, We and Wa.

Chain A of 3X42 is depicted in Figure 6A; the presence of many Na (purple) and Br (dark red) ions is clearly noticeable. Clustering with DBSCAN does not find any highly conserved clusters of Br ions; thus, we easily determine that they are not ordinarily present in AGAO and are a direct consequence of the experimental protocol. We can readily identify the most important metal ion in the structure, the Cu ion with a corresponding 93% conservation cluster at position 21.29, −3.40, 19.21 Å. Since 3X42 represents a *holo* structure, we can compare the binding site of Cu in the actual structure with the clusters predicted by the MADE approach, as shown in Figure 6B. The brown sphere represents the Cu from 3X42, and the red sphere in almost the same position, 0.17 Å away, marks the Cu location predicted by the MADE approach. The Cu ion in AGAO displays a distorted square pyramidal geometry; the Cu is coordinated by His-592, His-431, and His-433^80,81^ at a distance of around 1.9, 2.0, and 2.1 Å, respectively, from the Cu position predicted by the MADE approach (and around 2.0 Å from the Cu in 3X42).

Besides the Cu ion, three conserved water molecules are included in the binding site. Wa and We form the axial and equatorial ligands of the distorted square pyramidal geometry at the Cu ion. W1 interacts with Wa and Tyr-284 residues. The locations of the conserved water molecules in the 3X42 structure are shown as red cross markers and the clusters predicted by the MADE approach as blue cross markers. W1 is predicted at position 19.18, −3.72, 15.40 Å (0.26 Å away from the W1 of 3X42) with 99% conservation, Wa is predicted at position 20.39, −2.06, 17.42 Å (0.12 Å away from Wa of 3X42) with 73% conservation, and We is predicted at 21.6, −1.41, 19.74 Å (0.13 Å away from We of 3X42) with 77% conservation. Hydrogen bonds are denoted with yellow dotted lines.

This example shows that the MADE approach can distinguish between, e.g., solvent or solvent constituent ions part of the experimental procedure and the highly conserved structural protein ions effectively. The location of the Cu ion and conserved waters in the 3X42 binding site is virtually identical to the location predicted by the MADE approach.

#### Cytochrome *c*’ Predicted Structure

Here, we present another possible use case of the MADE approach, locating the cofactors in protein structures predicted by tools such as AlphaFold2.^26−28^ Protein structure prediction tools often do not include the (ionic) cofactors in their predicted structures, which also holds true for AlphaFold2.^29,30^

We have decided to investigate the cytochrome *c*’ protein, which is present in a variety of denitrifying, nitrogen-fixing, and photosynthetic bacteria. It bears some resemblance to the mitochondrial c class of proteins: they are polypeptides consisting of around 120–130 amino acid residues (≈14 kDa), and they contain a Heme C group bound by two Cys-thioester bonds with a common Cys-X-Y-Cys-His motif. Unlike the mitochondrial cytochrome *c* proteins, the cytochrome *c*’ structure does not provide a sixth ligand for the Heme C group. Instead, it can bind small neutral ligands such as CO and NO at the vacant sixth coordination site. The functional role of cytochrome *c*’ is not fully understood, and its physiochemical properties suggest a possible role in electron transfer. It has been proposed that in denitrifiers, cytochrome *c*’ has a role in NO-transfer mediation.^82−84^

We analyzed the cytochrome *c*’ structure from *Achromobacter xylosoxidans*, described by the UniProt ID P00138. We used the structure predicted by AlphaFold2, which is also described in the RSCB PDB with CMS ID AF_AFP00138F1. Using the MADE approach, with a 95% sequence identity cutoff, we locate a set of 51 experimental structures homologous to the predicted structure with 1 chain per structure for a total of 51 homologous chains.

**Figure 7:**
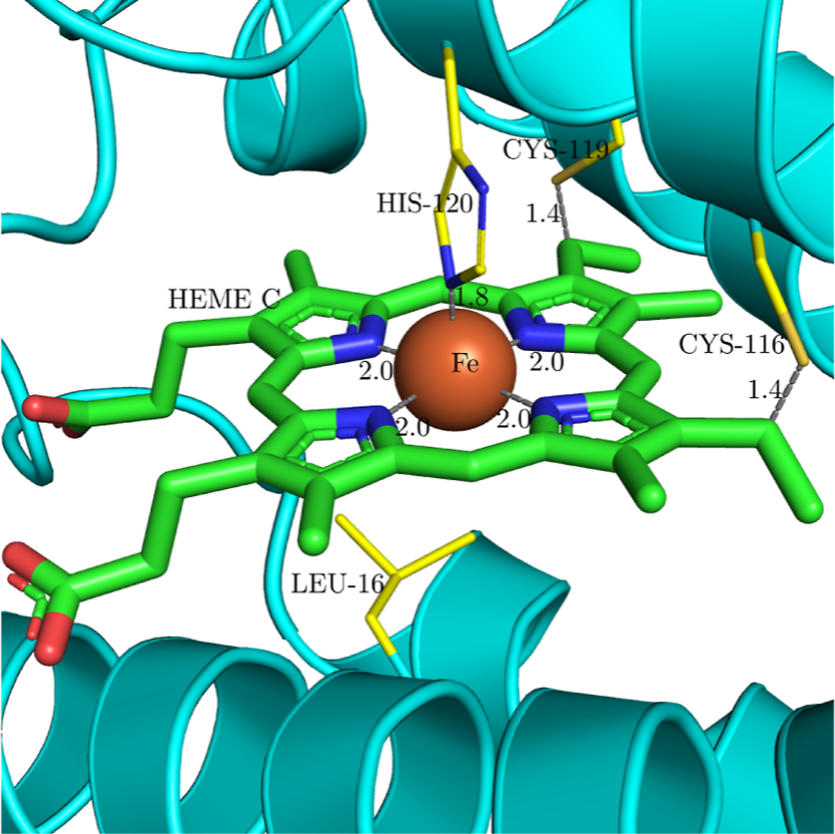
Cytochrome *c*’ structure predicted by AlphaFold2 with UniProt ID P00138 (RCSB PDB CMS ID AF_AFP00138F1). The full Heme C structure was predicted by the MADE approach. Heme C is covalently bound via thioester bonds with Cys-116 and Cys-119, the Fe ion in Heme C has five ligands, four from nitrogen atoms of the porphyrin ring, and one from the polypeptide, where it is coordinated by the nitrogen of His-120. Numbers represent coordinate bond distances in Å.

Superimposing the homologous chains upon the predicted structure and clustering with DBSCAN, we locate clusters for each of the atoms in the Heme C. The clusters of the carbon and nitrogen backbone of the Heme C are very well conserved, with conservations between 94 and 100%. The Fe ion of the heme is located with 98% conservation. The oxygen atoms on the carbonyl groups are less conserved with one group having a conservation of 90% and the other a much lower conservation of 62%. Figure 7 shows the binding site of the Heme C structure in the cytochrome *c*’ predicted by AlphaFold2. Heme C is covalently bound to the Cys-119 and Cys-116 with thioester bonds. In this structure, the bond distance between the cysteine residues and the Heme C predicted by the MADE approach is 1.4 Å, which is somewhat lower than in the experimental structures, where the thioether bond distance is typically around 1.8 Å.^82,84^ The Fe His-120 coordination bond is around 1.8 Å in the predicted structure and slightly longer, around 2.0 Å, in the experimental structures.^82,84^ The distances between the predicted and experimental structures differ because the His and Cys side chains are in a slightly different positions in the AlphaFold predicted structure, which leads to incorrect bond distances. Coordination bond distances within the Heme C itself are consistent between the predictions from the MADE approach and experimental structures, around 2.0 Å. The vacant sixth coordination site of the Fe ion is covered by Leu-16.

This example serves to further demonstrate the versatility of the MADE approach; it can also be applied to computationally predicted protein structures since these generally do not include the often crucial cofactors. Alternative tools such as AlphaFill^29^ already exist and apply sequence and structure similarity to “transplant” small molecules and ions to protein models from experimentally determined structures. We have also expanded on the generality of the MADE approach; in this example, we have predicted a full Heme cofactor, not just the Fe ion crucial for its structure and function.

### Comparison of Superposition Methods

In the previous section, we demonstrated the devised MADE methodology. Here, we will compare the results of the MADE approach using different superposition techniques in an effort to generalize the method. We will compare the superposition methods implemented in the MADE plugin (*align* and *super* commands in PyMOL, TM-align, DeepAlign, GANGSTA+, and ProBiS). The selected methods encompass the majority of the main classes of superposition techniques and are described in more detail in the Methods section.

The superposition methods available in the MADE plugin are varied, for example, DeepAlign and PyMOL *align* represent sequence-dependent methods, while the others are sequence independent. GANGSTA+ heavily focuses on the alignment of SSEs, while most other methods consider the whole protein backbone. ProBiS aligns the proteins only locally based on the similarity of parts of the protein surfaces, while the remaining methods perform global superposition.

**Figure 8:**
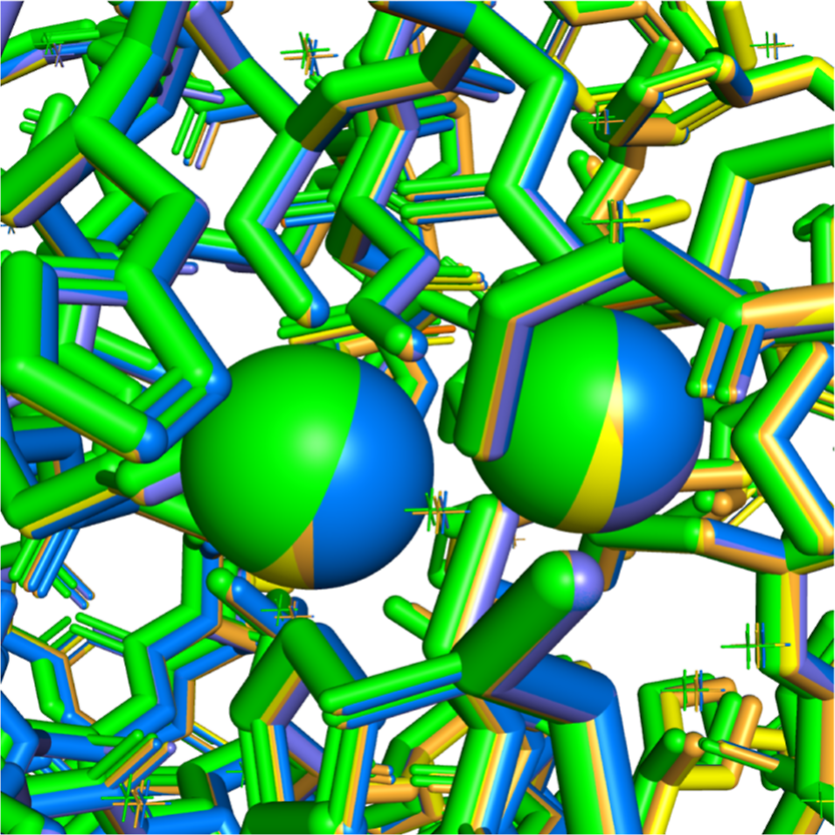
Example of the results of different superposition algorithms: chain A of 2DOO IMP-1 metallo-β-lactamase superposed onto 6JED (not shown) using various superposition algorithms. ProBiS (green), TM-align, DeepAlign, and GANGSTA+ (shades of yellow and orange) as well as PyMOL *align* and *super* (shades of blue).

Figure 8 shows the result of applying all of the above superposition methods on the same system, superimposing chain A of 2DOO^85^ onto chain A of 6JED,^86^ where both structures represent IMP-1 metallo-β-lactamases (containing 2 Zn ions). Both PyMOL superposition methods yield similar results; the chains superposed with PyMOL are depicted with shades of blue in Figure 8. TM-align, GANGSTA+, and DeepAlign also result in a very similar superposition in this particular example; they are shown in shades of yellow and orange. Finally, ProBiS, depicted in green, gives results that slightly differ from the others in this particular example.

In this section, we will compare the metal binding site prediction results of the MADE approach when using different superposition methods. Our main focus will be comparing the conservation of clusters determined by different superposition methods, as well as the root-mean-squared deviation (RMSD) of the positions of cluster constituents. In this section, we will use *holo* protein structures, which allow us to compare the native positions of the metal ion from PDB records with the positions of the corresponding clusters predicted by the MADE approach. We will also compare the results when superimposing a set of homologous structures upon the whole protein chain of the query and when focusing the superposition on a specific smaller binding site.

The first structure we used to compare the superposition methods represents a zinc finger, PDB accession code 1A1H.^87^ More specifically, it belongs to a modified three-finger Zif268 peptide–DNA complex and consists of three C_2_H_2_ zinc finger domains containing in total three Zn ions. We applied the MADE plugin to form a cluster of homologous structures at 95% sequence identity, which contained 16 complexes with a total of 17 superimposed protein chains.

First, we used the compare whole chain option of the MADE plugin to superimpose the homologous structures upon the whole chain A of 1A1H. All studied superposition methods find all the three expected Zn ion clusters, almost always with a 100% cluster conservation. ProBiS represents the only exception, which finds only 2 100% conserved Zn ion clusters and the third cluster with a 71% conservation. Table 1 displays the RMSD in the coordinates of the superimposed Zn ions within the clusters: the difference between the superposition methods is slight with the RMSD of around 0.15 Å. ProBiS again stands out with a larger RMSD of around 0.24 Å. The other metric collected in Table 1 is the average distance of the Zn ion in the original PDB structure to the cluster determined by the MADE approach, *d*_c-ZN_. We can notice some differences in *d*_c-ZN_ between superposition methods, in this example, the PyMOL algorithms seem to yield clusters closest to the experimentally determined Zn ions. It is worth mentioning that *d*_c-ZN_ is likely not the best measure of how well a given superposition algorithm performs, but it is an important indicator nonetheless.

**Table 1:** Average RMSD of Coordinates of Zinc Ions within Clusters and the Average Distance from the Native Zn Ion in the Original PDB Structure to the Cluster Determined by the MADE Plugin, *d*_c-ZN_, for Different Superposition Methods^a^

Superposition tool	RMSD [Å]	*d*_c-ZN_ [Å]
PyMOL *align*	0.147	0.190
PyMOL *super*	0.150	0.196
TM-align	0.144	0.243
DeepAlign	0.144	0.247
GANGSTA+	0.147	0.256
ProBiS	0.237	0.282

a The results given are for the zinc finger with PDB ID 1A1H using whole chain analysis.

We also applied the MADE plugin to analyze the 1A1H complex using local superposition, superimposing the homologous protein structures only upon the amino acid residues surrounding the binding site. In this case, we present averaged results over local superpositions around each of the three Zn ions. Local superposition means that the MADE plugin superimposes a set of homologous protein structures only using the area around the selected binding site. ProBiS represents the only tool, which specializes in local superposition; it even possesses an explicit local binding site superposition option, which the MADE plugin utilizes. Local superposition with the remaining superposition methods is achieved by superimposing the homologous chains only on a fragment of the query protein structure around the binding site. Local superposition can lead to better superposition in the specific area and consequently to lower RMSD values and higher conservation of clusters. This is useful if we are interested only in species around a given binding site and not in the rest of the protein structure. We, therefore, propose a twofold approach using whole chain superposition to identify putative binding sites and subsequent local superposition to refine the results for the binding sites of interest.

The results when using the MADE approach with each of the three Zn ions of 1A1H as binding sites and averaging the results for each of the superposition methods are presented in Table 2. We notice that the average RMSD and *d*_c-ZN_ are indeed lower than with whole chain superposition. The only exception is GANGSTA+, where we see significantly worse results. GANGSTA+ also finds clusters of Zn ions with 59% average conservation, unlike all the remaining superposition methods which find all the clusters 100% conserved. GANGSTA+ is based on finding alignments between SSEs. This indeed seems to cause GANGSTA+ to be less reliable when superimposing small structures with not many SSEs, which is often the case when using local superposition. Selecting a larger portion of the query to perform the local superposition upon can thus lead to better results with GANGSTA+. There are no significant differences between the remaining methods. ProBiS which performed the worst with whole chain superposition yields similar results to the other methods using local superposition. This example also demonstrates the usefulness of directing the superposition to a smaller part of the whole system. The resulting superposition is better in the selected area, and consequently, the clusters found by the MADE approach exhibit a smaller RMSD and a higher conservation. This does not mean that local superposition is necessarily better than whole chain superposition; to run local superposition, we need additional information about the system, namely, which binding site to use it for. Meanwhile, whole chain superposition gives results for the whole protein chain, albeit often with worse cluster conservation and RMSD.

**Table 2:** Average RMSD of Coordinates of Zinc Ions in Identified Clusters and the Average Distance from the Zn Ion in the PDB Original Structure to the Cluster Determined by the MADE Approach, *d*_c-ZN_, for Different Superposition Methods^a^

Superposition tool	RMSD [Å]	*d*_c-ZN_ [Å]
PyMOL *align*	0.086	0.150
PyMOL *super*	0.104	0.151
TM-align	0.093	0.179
DeepAlign	0.094	0.184
GANGSTA+	0.202	0.931
ProBiS	0.103	0.141

a The results are for zinc finger with PDB ID 1A1H using local superposition.

To further compare the superposition methods implemented in the MADE plugin, we used another *holo*-enzyme structure, this time of a class B1 metallo-β-lactamase. This particular structure represents an IMP-1-type MβL with PDB accession code 6JED deposited in a recent study (ref (86)) on MβL inhibitors. 6JED represents a structure with a single protein chain (A), which contains Zn ions in both the 3H and DHC binding sites.^86^ Using the MADE plugin, we find a set of 27 structures sharing at least 95% sequence identity with 6JED. Among these, many possess multiple homologous chains for a total of 74 superimposed protein chains.

In Table 3, we see the average conservation, RMSD, and *d*_c-ZN_ of both Zn ions using whole chain superposition. *d*_c-ZN_ is the average distance from the cluster predicted by the MADE approach to the corresponding Zn ion in the 6JED PDB structure. The results again show that ProBiS performs worse than the remaining superposition methods when using whole chain analysis corresponding to the local superposition focus of ProBiS. In this case, the PyMOL superposition algorithms again perform the best.

**Table 3:** Average RMSD of Coordinates of Zinc Ions in the Clusters and the Average Distance from the Zn Ion in the Original PDB Structure to the Cluster Determined by the MADE Approach, *d*_c–ZN_, for Different Superposition Methods^a^

Superposition tool	Conservation	RMSD [Å]	*d*_c-ZN_ [Å]
PyMOL *align*	0.91	0.226	0.131
PyMOL *super*	0.91	0.226	0.131
TM-align	0.84	0.207	0.134
DeepAlign	0.84	0.224	0.118
GANGSTA+	0.82	0.213	0.135
ProBiS	0.78	0.302	0.189

a Results are for IMP-1 MβL with PDB ID 6JED using whole chain superposition.

On the other hand, the results when using both Zn ions as binding sites for local superposition from Table 4 again show somewhat less scattered clusters with lower RMSD and *d*_c-ZN_ and with higher conservation. The notable exception is again GANGSTA+, which again performs much worse with local superposition. In the case of 6JED, ProBiS does not only perform similarly to other methods but actually gives the best results with local superposition.

**Table 4:** Average RMSD of Coordinates of Zinc Ions in the Clusters and the Average Distance from the Zn Ion in the Original PDB Structure to the Cluster Determined by the MADE Approach, *d*_c-ZN_, for Different Superposition Methods^a^

Superposition tool	Conservation	RMSD [Å]	*d*_c-ZN_ [Å]
PyMOL *align*	0.912	0.206	0.126
PyMOL *super*	0.909	0.217	0.189
TM-align	0.797	0.220	0.138
DeepAlign	0.902	0.205	0.128
GANGSTA+	0.237	0.242	0.796
ProBiS	0.926	0.181	0.123

a Results are for IMP-1 MβL with PDB ID 6JED using local superposition.

Summing up the results of the two examples comparing different superposition methods, in most cases, they all yield similar results when used with the MADE approach. Superposition with GANGSTA+ is suboptimal when using local superposition. Since GANGSTA+ is based on the superposition of SSEs using local superposition with smaller binding sites containing fewer residues, and consequently fewer SSEs likely leads to worse results. This is supported by the fact that GANGSTA+ also struggles with the superposition of small structures even when using whole chain superposition. For example, using GANGSTA+ on PDB ID 1RIK (the zinc finger from the previous section) which consists of 29 residues results in only around 40% cluster conservation (the remaining methods give around 80% conservation). GANGSTA+ works well on queries with more than a few SSEs, but we recommend against using it on very small queries or for local superposition. When superposing whole chains, ProBiS yields slightly worse results than the remaining superposition methods, but ProBiS excels when performing local superposition. The ProBiS algorithm identifies the solvent-accessible surface of the protein and presents it as a protein graph, a structure of vertices and edges that takes into account both the physicochemical and geometrical properties of the surface. ProBiS then applies a fast maximum clique algorithm^52^ to perform a local, surface-oriented comparison of the protein surface graphs.^51,54^ This makes ProBiS an excellent choice for performing local superposition with the MADE approach, as demonstrated by the above examples. ProBiS is also the only tool with inbuilt support for local superposition, and we have shown that it yields similar or even better results than the alternative methods. Therefore, we recommend using ProBiS when performing local superposition; alternative methods are better suited when superposing whole chains.

Apart from the above examples, we found no significant differences between the results of the MADE approach when using different superposition methods. This demonstrates that the MADE methodology is robust since it produces results independent of the superposition method.

When choosing, which superposition method to use, one first needs to consider if one wants to use whole chain superposition or local binding site superposition. For local superposition, we recommend the ProBiS algorithm since this represents its specialty. ProBiS applies a parallelized, fast maximum clique algorithm to locate similar local patches in the query and superposed protein structures. ProBiS is the only algorithm that features an inbuilt option to select only residues within a certain radius around a given ligand. Based on these features, as well as the ProBiS algorithms’ focus on local superposition, ProBiS is our clear preferred option for local superposition. If we are superimposing whole chains, we recommend choosing a different algorithm than ProBiS.

Out of the superposition methods included in this study, PyMOL *align* and DeepAlign can be classified as sequence dependent; they take the amino acid sequence into account when performing the superposition. Because of their sequence-dependent nature, we recommend using them with proteins possessing a relatively high sequence identity. On the other hand, TM-align, GANGSTA+, and PyMOL *super* all belong to sequence-independent superposition algorithms. GANGSTA+ represents a tool that focuses on secondary structure alignment; it can perform non-sequential alignments ignoring the loops connecting the SSEs. We recommend its application in cases when we want a focus on the alignment of α-helices and β-sheets: for example, when structures have poorly defined loops connecting the SSEs. We recommend using TM-align and PyMOL *super* for proteins with a lower sequence identity due to their sequence-independent nature.

**Figure 9:**
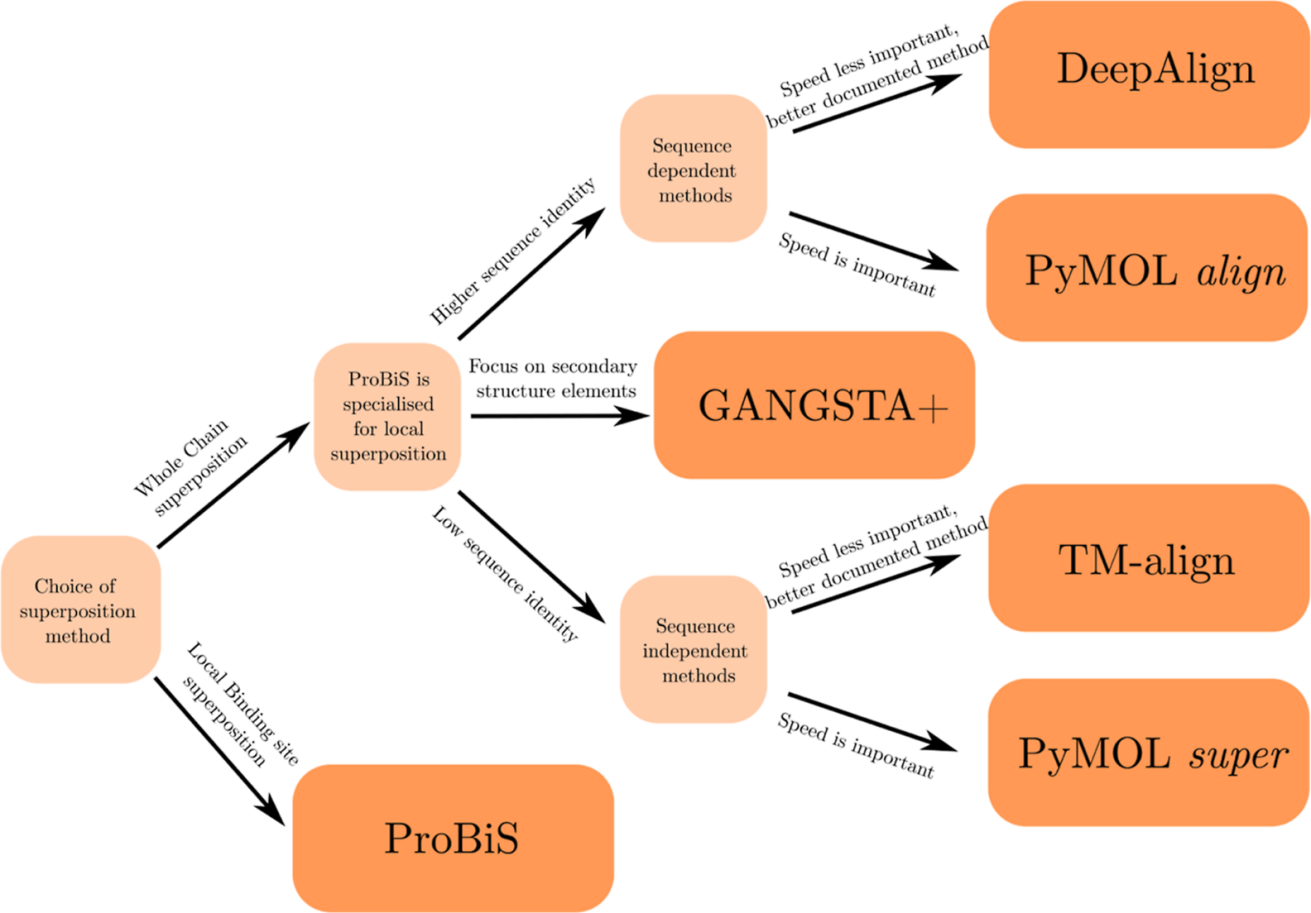
Diagram of our recommendations for superposition method selection. Use ProBiS for local superposition. DeepAlign and PyMOL *align* are sequence dependent; we recommend using them with high sequence identity proteins. GANGSTA+ can align proteins non-sequentially and focuses on secondary structure alignment; we recommend using it on systems where it is advantageous (poorly defined loops between SSEs etc.). We recommend using TM-align and PyMOL *super* on lower sequence identify proteins. The PyMOL algorithms work fastest in the MADE plugin, apply them if speed is important (many chains to superimpose), and the remaining methods are, however, better documented in the scientific literature.

Yet, another factor to consider is the calculation time. In our experience, the PyMOL algorithms provide the fastest calculations in the MADE plugin. A contributing factor to why the PyMOL algorithms perform faster is the fact that the whole process takes place within PyMOL; the plugin does not need to write and read data as well as run executables for the superposition algorithms. Additional information regarding superposition speed is provided in the Supporting Information. Thus, we recommend using PyMOL algorithms, if the time taken for the superposition represents a big factor. If the time taken for superposition is not a large concern, we recommend using one of the remaining algorithms since they are much better documented in the scientific literature,^48−51^ even though PyMOL algorithms yield similar results. A schematic diagram of our recommendation for superposition method selection is presented in Figure 9. We have to stress that these represent only general guidelines based on the types of superposition methods and on our experience with their use. In general, which method gives the best results changes from system to system, and as we have shown (with a few notable exceptions), all implemented methods yield similar results.

## Conclusions

In this article, we have introduced a novel method for analyzing different species present in protein structures, called the MADE approach. We have focused on the metal binding site prediction capabilities of this approach.

The MADE method applies experimental structures of homologous protein complexes to analyze and detect areas where a certain species is present at a similar location among many homologous structures. The idea behind the approach comes from a previous toolset for identifying conserved water molecules, ProBiS H_2_O (MD).^31,32^ The approach is implemented as the MADE plugin and is freely available for the PyMOL molecular visualization tool.

The first step in the MADE approach represents identifying structures homologous to a query protein. The set of homologous structures is then superimposed upon the query protein. In this work, we have considered several algorithms for protein superstition (the *align* and *super* commands of PyMOL, ProBiS, GANGSTA+, DeepAlign, and TM-align). We have shown the robustness of the MADE approach because the selection of the superposition method does not have a significant effect on the obtained results. We have also provided general guidelines about which superposition method to use based on the features of the superposition methods and on our experience working with the MADE approach.

The MADE approach identifies areas with a high density of a particular species. Such locations are structurally/mechanistically important to the query protein and merit further analysis. To locate such dense areas from the data about the atom positions in the superimposed structures, we employ a clustering algorithm 3D-DBSCAN. The main focus of this article is devoted to the MADE approach as a tool for predicting the positions of metal binding sites in *apo* protein structures. We have shown that the MADE plugin can successfully identify highly conserved clusters of the appropriate metal ion in the correct position in the binding site of several *apo* structures of well-studied metalloproteins. A significant advantage of the MADE approach lies in its generality since it can be applied to any species, not just metal ions, for example: conserved water molecules, experimental artifacts, or other types of non-protein and protein atoms. Conserved water molecules are of particular interest; in this article, we have demonstrated that besides detecting the locations of metal ions in proteins, the MADE methodology can also simultaneously reveal the location of conserved water molecules in these metal binding sites. Conserved water molecules can indeed be crucial for describing the coordination of the metal ions,^75,79^ and identifying them gives the MADE approach an advantage over the existing metal detecting methods. We have also demonstrated that the MADE approach can work on structures predicted by computational methods, such as AlphaFold,^26−28^ that do not include cofactors in the structure.

Critical for the MADE approach is its reliance on experimental data, and we postulate that its relevance will increase with additional experimental structures. This trend is especially evident with recent advances in cryo-EM^23,24,88^ since more and more structures are determined using this experimental technique.

In the future, data for density analysis could also be obtained by using theoretical approaches such as MD simulations^32,89−91^ facilitating the applicability of the MADE approach to less-investigated systems. Such an extension has already been performed with good results for conserved water identification with MD simulations.^32,92^ In the future, if structure prediction tools such as AlphaFold evolve to include cofactors, they may also provide a means for our approach to obtain additional structural data. We also advocate the use of MD simulations on identified conserved water molecules for further structural optimization and for a critical study of dynamic water networks.

## Data Availability

The MADE plugin for the PyMOL molecular visualization software is available in a GitLab repository: https://gitlab.com/Jukic/made_software. The plugin is supported on the Windows and Linux operating systems (Support might be extended to macOS in the future), it is written for python 3 and PyMOL v2.x. Detailed application and installation instructions are included in the MADE plugin tutorial on the GitLab repository. The structural data analyzed with the MADE plugin in this study was obtained from the RCSB PDB. A detailed list of which homologous PDB structures were used is available in the Supporting Information, as well as a custom_clusters.txt file applicable by the MADE plugin containing the same information. This should allow for the recreation of all the results presented in this study.

## Supporting Information Available

Additional details about the analyzed systems and information about the computation speed of the MADE plugin (PDF)
Custom_complexes.txt (.txt): A file readable by the MADE plugin containing the sets of homologous complexes used in the study (TXT)
