# Supplementary material for: Identifying Metal Binding Sites in Proteins Using Homologous Structures, the MADE Approach
Source: J Chem Inf Model. 2023 Aug 9;63(16):5204–19. doi: 10.1021/acs.jcim.3c00558 (PMC10466382; doi:10.1021/acs.jcim.3c00558)
Supplement: Supplementary file 1 — ci3c00558_si_001.pdf [file ci3c00558_si_001.pdf]

Supporting Information: Identifying Metal Binding Sites in Proteins Using Homologous Structures, the MADE Approach

Vid Ravnik,[†] Marko Jukič,[*,†,‡] and Urban Bren[*,†,‡,¶]

†*Faculty of Chemistry and Chemical Engineering, University of Maribor, Smetanova ulica 17, SI-2000 Maribor, Slovenia*

‡*The Faculty of Mathematics, Natural Sciences and Information Technologies, University of Primorska, Glagoljaška 8, SI-6000 Koper, Slovenia*

¶*Institute for Environmental Protection and Sensors, Beloruska ulica 7, SI-2000 Maribor, Slovenia*

E-mail: marko.jukic@um.si; urban.bren@um.si

Additional examples

Here we provide two additional examples of the application of the MADE approach to metal binding in proteins to further demonstrate the applicability for some metal ions not considered in the main manuscript.

Calpain

Calpains are calcium-regulated cysteine proteases that are abundant in the cytoplasm of mammalian cells. We have analyzed an *apo* structure of the Ca^{2+} binding domain (dVI) of rat m-calpain with PDB ID 1AJ5.[S1] The Ca^{2+} binding sites represent helix-loop-helix structures, EF-hand motifs.[S1]

Using a 95% sequence identity we find 7 complexes homologous to 1AJ5: 3BOW, 1DF0, 3DF0, 1AJ5, 1NP8, 1DVI, 1U5I, and 6QLB, for a total of 12 chains superimposed upon the query protein chain A of 1AJ5.

Using DeepAlign as the superposition method, clustering with DBSCAN finds 4 clusters of Ca^{2+} ions with 62% conservation. The lower conservation is not due to bad superposition, but because some of the structures do not contain bound ions, while some others contain bound cadmium ions. The structure of the calpain dVI domain with the predicted Ca^{2+} clusters (green spheres) is shown in Figure S1. We can also locate clusters of water molecules, the EF2 and EF3 binding sites are canonical EF-hand motifs and the Ca is coordinated with 1 conserved water molecule.[S1] The EF1 binding site is different, the Ca^{2+} is coordinated by two water molecules. The EF4 binding site only binds Ca^{2+} at higher concentrations, the bound Ca^{2+} is also coordinated by two conserved water molecules.[S1] The MADE approach locates W4, W5, and W6 with 62% conservation, W2 with 54% conservation, as well as W1 and W3 with 46% conservation. In this particular example, the side chains of the EF hands of the *apo* protein have different orientations from the *holo* structure, leading to the distances between the predicted Ca^{2+} and the coordinating residues being larger than expected.

Phosphodiesterase

Phosphodiesterase 4 (PDE4) represents a major family of enzymes whose function involves the hydrolysis of cyclic adenosine monophosphate (cAMP). They are highly expressed in many different tissues, including the brain, smooth muscles, cardiovascular tissues, immunocytes, and keratinocytes.[S2]

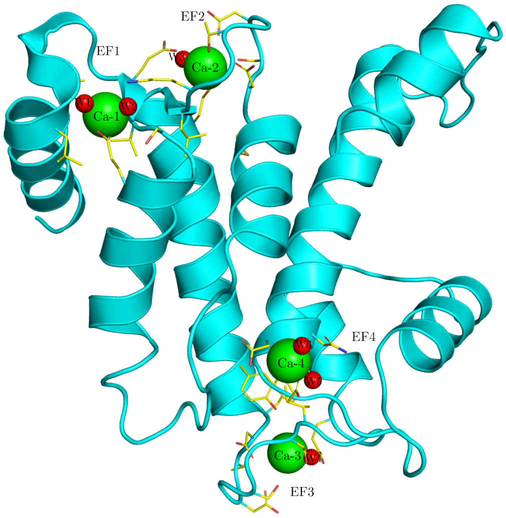

Figure S1: Ca^{2+} binding domain (dVI) of rat m-calpain with PDB ID 1AJ5[S1] analyzed with the MADE approach. The four predicted Ca^{2+} bindings sites are shown with green spheres, while the predicted coordinating water molecules are depicted as small red spheres.

We have analyzed a *holo* structure of a human phosphodiesterase 4D with PDB ID 3IAD[S3] using the MADE approach. With a 95% sequence identity cutoff, we identify 94 complexes homologous to 3IAD: 1XOM, 1ZKN, 1Y2C, 1TBB, 1Y2K, 1Y2E, 1Y2D, 1PTW, 1XOQ, 1TB7, 1XOR, 1MKD, 1OYN, 1XON, 1Y2B, 1Q9M, 2FM0, 2QYN, 2FM5, 2PW3, 3G4I, 3G4G, 3IAK, 3K4S, 3G4L, 3SL3, 3G58, 3G4K, 3SL6, 3SL4, 3SL5, 3V9B, 4OGB, 5K1I, 4W1O, 4WCU, 3SL8, 5WQA, 5LBO, 5WH5, 6F6U, 5K32, 6F8R, 6F8T, 5TKB, 6BOJ, 6AKR, 6F8V, 6F8U, 6F8W, 6FDC, 6F8X, 6FE7, 6FEB, 6FET, 6FDI, 6FTA, 6FT0, 6IAG, 6FTW, 6HWO, 6FW3, 6IBF, 6IMI, 6IMD, 6IM6, 6IMB, 6INK, 6IMT, 6IMR, 6IMO, 6INM, 6IND, 6KK0, 6KJZ, 6LRM, 6NJI, 6NJH, 6NJJ, 6ZBA, 7A8Q, 7A9V, 6RCW, 7AAG, 7ABD, 7AB9, 7ABE, 7ABJ, 7AY6, 7B9H, 7CBQ, 7CBJ, 7W4X, and 7W4Y. Superposing 269 chains with DeepAlign upon the query protein chain A of 3IAD, we find two highly conserved clusters of metal ions, a cluster of Zn^{2+} with 100% conservation (at position 17.824, 1.741, 26.347 Å) and a cluster of Mg^{2+} with 83% conservation (at position 16.617, -0.506, 23.517 Å). We also locate 6 clusters of conserved water molecules, all with 75% or more conservation, that are involved in the metal binding. The binding site with the predicted ion and water clusters is shown in Figure S2. Since the 3IAD structure represents a *holo* enzyme, we can compare

the positions of the predicted ions and water molecules with the ions and water molecules present in the experimental structure. The experimental Mg^{2+} ion (green sphere in Figure S2) is 0.5 Å distant from the mean cluster position (brown sphere), while the distance between the experimental (light purple sphere) and predicted Zn^{2+} ions is much shorter, 0.1 Å. Two of the six identified water clusters (small red spheres) have corresponding water molecules in the 3IAD (chain A) structure (small yellow spheres), the distance between the predicted and experimental position is 0.5 Å for W1 and 0.2 Å for W4, respectively. The scientific literature[S2] tells us that the binding of both the zinc and magnesium ions is octahedral, with the zinc being coordinated by two histidine and two aspartate residues (His-330, His-366, Asp-367, and Asp-484) and the magnesium by only a single bridging aspartate (Asp-367). The vacant coordination positions are filled by a bridging water molecule and the remaining water molecules. The water clusters predicted by our approach are in excellent agreement with this statement, W1 is the bridging water coordinating both ions, W6 coordinates only the Zn^{2+} ion while W2, W3, W4, and W5 coordinate the Mg^{2+} ion. This example clearly demonstrates that even in *holo* structures, the MADE methodology can be applied to fill in the missing ligands (most commonly conserved water molecules) involved in the metal binding site.

Analyzed System Details

Here we present some additional details about the systems analyzed with the MADE plugin throughout the article. The results presented in the article were obtained around March 2022, the structures and sequence similarity clusters reflect the state of PDB data from that time. We know for a fact at least 1 of the structures, 1RIK, has a vastly different sequence similarity cluster at 30% sequence identity at the time of writing (March 2023), only containing 4 homologous structures. The results presented in the article can still be recreated in the MADE plugin by using a custom cluster of complexes, a file (custom_complexes.txt,

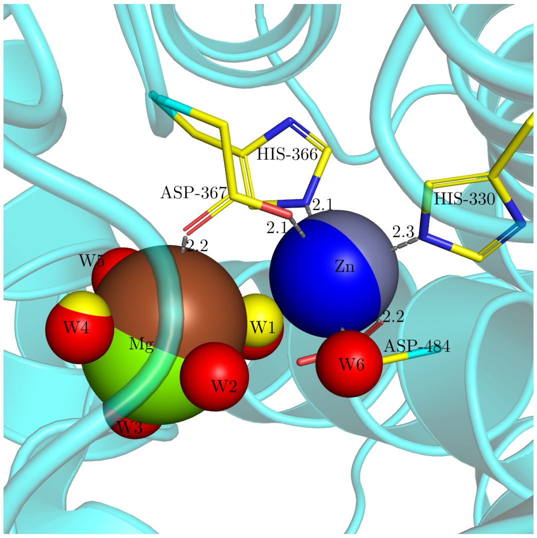

Figure S2: The binding site of the human phosphodiesterase 4D with PDB ID 3IAD[S3] analyzed with the MADE approach, the experimental positions of the Mg^{2+} (green sphere) and Zn^{2+} (light purple sphere) are visible, as well as the corresponding Mg^{2+} (brown sphere) and Zn^{2+} (blue sphere) clusters predicted by the MADE approach. The MADE approach also predicts 6 conserved water molecules involved in the binding site (small red spheres), two of these are also present in the experimental structure (small yellow spheres). Numbers denote coordinate bond distances in Å.

readable by the MADE plugin) with the sequence similarity complexes applied in this study, which is also provided in the Supplementary Information.

Zinc Finger, PDB ID 1RIK

1RIK,[S4] is a zinc finger, the structure includes 1 protein chain (A) with 29 residues. We find 18 PDB IDs in 30% sequence identity cluster of 1RIK: 2KVF, 1XF7, 6PV0, 6UCP, 6UCO, 6PV1, 6PV2, 1SP2, 1VA2, 6PV3, 5US3, 1SP1, 1RIK, 1VA3, 1ARE, 1ARD, 1ARF and 1ZNM. We superpose 17 protein chains upon chain A of 1RIK with DeepAlign. Clustering with 3D-DBSCAN reveals 1 cluster with 16 Zn ions, 89% conservation, located in the C_2H_2 binding site of 1RIK.

Manganese Superoxide Dismutase, PDB ID 3OT7

3OT7 is an *apo* structure of an *E. coli* manganese superoxide dismutase, the structure consists of 4 protein chains (A, B, C, and D) with 205 residues each. We find 13 PDB IDs in 95% sequence identity cluster of 3OT7:[S5] 1ZLZ, 1VEW, 1EN4, 1IXB, 1I0H, 1EN5, 3OT7, 1IX9, 3K9S, 1MMM, 1EN6, 1I08 and 1D5N. We superimpose 38 protein chains upon chain A of 3OT7 with DeepAlign and we identify the important clusters using 3D-DBSCAN: 2 clusters with 34 Mn ions, conservation 87%. The clusters are located in the binding sites of the A and B chains of 3OT7 2 clusters with 6 OH^- ions, conservation 15%. The clusters are located in the binding sites of the A and B chains of 3OT7, near the Mn ion clusters. 2 clusters with 29 H_2O, conservation 74%; their clusters are located in the binding sites of the A and B chains of 3OT7, in the same location as the OH^- clusters. It is worth mentioning that there are many additional water clusters with higher conservation, we find 85 water clusters with conservation of 74% or above.

Metallo β-Lactamase, PDB ID 3I0V

3I0V[S6] is a *apo* structure of a BCII metallo β-lactamase from *Bacillus cereus*. The 3I0V structure contains 1 chain (A) with 211 residues. We find 44 PDB IDs in a 95% sequence similarity cluster of 3I0V: 2UYX, 2BG2, 3KNS, 2BG8, 2BFZ, 1MQO, 4TYT, 6EUM, 1DXK, 2BFL, 1BVT, 3I15, 3I11, 2BC2, 3KNR, 6F2N, 3I13, 2M5C, 4C1H, 5JMX, 4C09, 1BC2, 2BFK, 4C1C, 2M5D, 5FQB, 5FQA, 2BGA, 3I0V, 3BC2, 6EWE, 2BG7, 3I14, 2BG6, 4NQ6, 4NQ4, 4NQ5, 3FCZ, 2NZE, 5W8W, 2NYP, 1BMC, 2NZF and 2NXA. The superposition of 61 protein chains upon chain A of 3I0V with DeepAlign, followed by clustering with 3D-DBSCAN reveals one cluster with 53 Zn ions (85% conservation), and another Zn cluster with 27 Zn ions (44% conservation). The clusters are located in the 3H and DCH binding sites of 3I0V respectively. We also find an important cluster of conserved water molecules in the binding site, with 36 waters (58% conservation). There are again many more water clusters with higher conservation, 80 clusters with 58% or higher conservation when using

whole chain superposition.

Copper Amine Oxidase, PDB ID 3X42

3X42[S7] is a copper containing amine oxidase, the structure contains 2 chains (A, B) with 620 residues each. In a 95% sequence similarity cutoff of 3X42, we find 87 PDB IDs: 2BT3, 2CFD, 1W4N, 1SII, 2CFW, 2CFK, 2CFG, 1W5Z, 1W6G, 2CFL, 1SIH, 1W6C, 3KII, 3KN4, 2CG0, 2CG1, 1RJO, 1IVV, 1UI7, 2CWV, 2YX9, 1IQY, 1AV4, 2ZL8, 3AMO, 1IQX, 1WMO, 1IVW, 2CWU, 2CWT, 7F8K, 2D1W, 1IVX, 1WMP, 2E2T, 1AVK, 1WMN, 1IVU, 1AVL, 1UI8, 1IU7, 2E2V, 2E2U, 3X42, 3WA2, 6L9C, 3WA3, 3X40, 5ZPL, 5ZP9, 5ZPC, 5ZPJ, 5ZP7, 5ZPA, 5ZPO, 5ZOU, 5ZPG, 3X3Y, 3X41, 5ZOX, 5ZP6, 5ZP8, 5ZPM, 5ZPR, 5ZOY, 5ZPH, 5ZPP, 5ZP4, 5ZPD, 5ZPK, 5ZPF, 5ZP1, 5ZPB, 5ZPN, 5ZP2, 5ZPT, 5ZPQ, 3X3X, 5ZPS, 3X3Z, 5ZP3, 5ZP0, 5ZPE, 5ZOW, 5ZOZ, 5ZPI and 5ZP5. Superposition (with PyMOL *align*) of 152 protein chains upon chain A of 3X42 and subsequent clustering with 3D-DBSCAN yield the following important clusters: 1 cluster of 142 Cu ions (93% conservation), located in the binding site of chain A, and another cluster with 75 Cu ions (49% conservation) in the binding site of chain B. 3 clusters of water molecules are of interest in the binding site of Cu, with 117, 111, and 151 members (77%, 73%, and 99% conservation).

Cytochrome c', predicted structure CMS ID AF_AFP00138F1

AF_AFP00138F1 is the Computed structure model of Cytochrome c' from *Achromobacter xylosoxidans*. The structure was predicted by AlphaFold2,[S8–S10] it contains 1 protein chain (A) with 127 amino acid residues. Since structures modeled by AlphaFold do not include the crucial cofactors we predicted these by applying the MADE approach. In a 95% sequence identity cluster of AF_AFP00138F1, we find 50 PDB IDs: 2XLD, 2XLV, 1E83, 2XLE, 2XL8, 2XLH, 1E84, 2XM0, 1E86, 2XLO, 1CGO, 2XLM, 1E85, 2XL6, 1CGN, 2XLW, 2XM4, 2YL0, 2YL1, 2YKZ, 2YL3, 2YLD, 2YL7, 3ZQV, 3ZTM, 3ZQY, 2YLG, 2YLI, 3ZTZ, 4CDV, 3ZWI, 4CDA, 4CDY, 4CIP, 4D4X, 4CJG, 4CJO, 4WGY, 4WGZ, 4D4N, 5AGF, 5JLI, 5JSL, 5JP7,

5JUA, 5JRA, 5JS5, 5JT4, 5JVE, 5NC0, and 5NGX. Superposition with DeepAlign reveals clusters of all 43 atoms from Heme C (residue name HEC), their conservation is listed in Table S1.

Table S1: The conservation of Heme C clusters identified by the MADE approach for the predicted structure of Cytochrome c'

HETATM type	Conservation [%]	HETATM type	Conservation [%]
HEC-CAA	100	HEC-C1B	98.1
HEC-CAB	100	HEC-C1C	98.1
HEC-CAC	98.1	HEC-C1D	98.1
HEC-CAD	96.2	HEC-O1A	90.4
HEC-NA	98.1	HEC-O1D	61.5
HEC-CBA	96.2	HEC-C2A	100
HEC-CBB	100	HEC-C2B	98.1
HEC-CBC	98.1	HEC-C2C	98.1
HEC-CBD	96.2	HEC-C2D	98.1
HEC-NB	98.1	HEC-O2A	90.4
HEC-CGA	96.2	HEC-O2D	61.5
HEC-CGD	94.2	HEC-C3A	98.1
HEC-ND	98.1	HEC-C3B	100
HEC-CHA	98.1	HEC-C3C	98.1
HEC-CHB	98.1	HEC-C3D	98.1
HEC-CHC	100	HEC-C4A	98.1
HEC-CHD	98.1	HEC-C4B	98.1
HEC-CMA	98.1	HEC-C4C	98.1
HEC-CMB	98.1	HEC-C4D	98.1
HEC-CMC	98.1	HEC-NC	98.1
HEC-CMD	98.1	HEC-FE	98.1
HEC-C1A	98.1		

Zinc Finger, PDB ID 1A1H

1A1H is a structure of a Zn finger DNA complex with 3 zinc finger domains. The structure contains a protein chain (A) with 87 residues and 2 DNA chains (B, C). We find 16 PDB IDs in a 95% sequence similarity cluster of 1A1H: 4R2D, 4R2A, 4R2C, 1AAY, 1A1J, 1JK2, 1A1I, 1A1L, 1A1H, 1A1F, 1JK1, 1A1G, 1A1K, 4X9J, 1P47 and 1ZAA. Superposition with various methods reveals 3 clusters of Zn ions.

Metallo β-lactamase, PDB ID 6JED

6JED is the *holo* structure of an IMP1 metallo β-lactamase, the structure contains 1 protein chain (A) with 221 residues. We find 27 PDB IDs in a 95% sequence similarity cluster: 4F6Z, 7XHX, 4F6H, 6ZYS, 5HH4, 5EWA, 5EV6, 6ZYR, 5EV8, 1WUP, 4C1F, 7DTN, 6LBL, 6JED, 4UAM, 1WUO, 1VGN, 1DD6, 7DTM, 1JJT, 4C1G, 6JKA, 5Y5B, 2DOO, 3WXC, 1JJE and 1DDK. Superposition with various methods reveals 2 clusters of Zn ions.

Superposition algorithm speed

We compared wall time for protein alignment of all the different superposition algorithms available in the MADE plugin, the results are presented in table S2. We used two complexes, 6JED and 3X42, which are the two largest systems used in the study as they encompass many large protein chains. In the case of 6JED the MADE plugin superimposes 74 chains of around 220 residues (26 kDa) each. 3X42 is a much larger system, the MADE plugin superposes 153 chains around 620 residues long (140 kDa), thus the superposition times in Table S2 are much longer. The results are wall times for just protein superposition obtained on a workstation with a 64 GB AMD Ryzen Threadripper 3960X 24-core processor. After superposition, the MADE plugin has to obtain all the clusters of heteroatoms which can also take some time. The clustering time is of course independent of the superposition method. In the case of 6JED, the MADE plugin finds around 13000 heteroatoms, and clustering takes approximately 3.3 s. The MADE plugin finds around 135000 heteroatoms in 3X42, therefore clustering takes much longer, around 117 s. It is worth mentioning that the majority of the clustering time is taken by the clustering of water molecules since they are usually by far the most abundant type of non-protein species.

We can see that the MADE plugin offers us fast calculation times; even for very large systems the calculation is completed within minutes. In most cases, we can expect results in much less than a minute. The PyMOL superposition methods perform the fastest, with

Table S2: The wall time for protein superposition of the different superposition methods in the MADE plugin

Superposition tool	Superposition time [s] (6JED)	Superposition time [s] (3X42)
PyMOL *align*	8.19	65.44
PyMOL *super*	8.40	67.73
TM-align	13.45	158.02
DeepAlign	22.75	831.51
GANGSTA+	37.65	391.86
ProBiS	9.96	140.63

ProBiS and TM-align being a bit slower. We have noticed ProBiS performing significantly slower (in comparison with the other methods) on less powerful computers, likely because its implementation is multi-threaded, and systems with fewer threads perform worse compared to the remaining methods. DeepAlign and GANAGSTA+ perform much slower than the other methods, this is especially true for larger systems.

References

(S1) Blanchard, H.; Grochulski, P.; Li, Y.; Arthur, J. S. C.; Davies, P. L.; Elce, J. S.; Cygler, M. Structure of a calpain Ca2+-binding domain reveals a novel EF-hand and Ca2+-induced conformational changes. *Nat. Struct. Biol.* **1997**, *4*, 532–538.

(S2) Furlan, V.; Bren, U. Insight into Inhibitory Mechanism of PDE4D by Dietary Polyphenols Using Molecular Dynamics Simulations and Free Energy Calculations. *Biomolecules* **2021**, *11*, 479.

(S3) Burgin, A. B.; Magnusson, O. T.; Singh, J.; Witte, P.; Staker, B. L.; Bjornsson, J. M.; Thorsteinsdottir, M.; Hrafnsdottir, S.; Hagen, T.; Kiselyov, A. S., et al. Design of phosphodiesterase 4D (PDE4D) allosteric modulators for enhancing cognition with improved safety. *Nat. Biotechnol.* **2010**, *28*, 63–70.

(S4) Liu, Y.; Liu, Z.; Androphy, E.; Chen, J.; Baleja, J. D. Design and characterization of

helical peptides that inhibit the E6 protein of papillomavirus. *Biochemistry* **2004**, *43*, 7421–7431.

(S5) Whittaker, M. M.; Lerch, T. F.; Kirillova, O.; Chapman, M. S.; Whittaker, J. W. Subunit dissociation and metal binding by Escherichia coli apo-manganese superoxide dismutase. *Arch. Biochem. Biophys.* **2011**, *505*, 213–225.

(S6) Gonzalez, J. M.; Buschiazzo, A.; Vila, A. J. Evidence of adaptability in metal coordination geometry and active-site loop conformation among B1 metallo-β-lactamases. *Biochemistry* **2010**, *49*, 7930–7938.

(S7) Murakawa, T.; Hamaguchi, A.; Nakanishi, S.; Kataoka, M.; Nakai, T.; Kawano, Y.; Yamaguchi, H.; Hayashi, H.; Tanizawa, K.; Okajima, T. Probing the catalytic mechanism of copper amine oxidase from Arthrobacter globiformis with halide ions. *J. Biol. Chem.* **2015**, *290*, 23094–23109.

(S8) Senior, A. W.; Evans, R.; Jumper, J.; Kirkpatrick, J.; Sifre, L.; Green, T.; Qin, C.; Žídek, A.; Nelson, A. W.; Bridgland, A., et al. Improved protein structure prediction using potentials from deep learning. *Nature* **2020**, *577*, 706–710.

(S9) Jumper, J.; Evans, R.; Pritzel, A.; Green, T.; Figurnov, M.; Ronneberger, O.; Tunyasuvunakool, K.; Bates, R.; Žídek, A.; Potapenko, A., et al. Highly accurate protein structure prediction with AlphaFold. *Nature* **2021**, *596*, 583–589.

(S10) Varadi, M.; Anyango, S.; Deshpande, M.; Nair, S.; Natassia, C.; Yordanova, G.; Yuan, D.; Stroe, O.; Wood, G.; Laydon, A., et al. AlphaFold Protein Structure Database: massively expanding the structural coverage of protein-sequence space with high-accuracy models. *Nucleic Acids Res.* **2022**, *50*, D439–D444.
